# A novel thermostable prokaryotic fucoidan active sulfatase PsFucS1 with an unusual quaternary hexameric structure

**DOI:** 10.1038/s41598-021-98588-3

**Published:** 2021-09-30

**Authors:** Maria Dalgaard Mikkelsen, Hang Thi Thuy Cao, Thomas Roret, Nanna Rhein-Knudsen, Jesper Holck, Van Thi Thanh Tran, Thuan Thi Nguyen, Vy Ha Nguyen Tran, Mateusz Jakub Lezyk, Jan Muschiol, Thinh Duc Pham, Mirjam Czjzek, Anne S. Meyer

**Affiliations:** 1 Protein Chemistry and Enzyme Technology Section, DTU Bioengineering, Department of Biotechnology and Biomedicine, Technical University of Denmark, 2800 Kgs Lyngby, Denmark; 2 NhaTrang Institute of Technology Research and Application, Vietnam Academy of Science and Technology, 02 Hung Vuong Street, Nhatrang, 650000 Socialist Republic of Vietnam; 3 Sorbonne Université, CNRS, Integrative Biology of Marine Models, Station Biologique de Roscoff, 29680 Roscoff, France

**Keywords:** Biochemistry, Biotechnology

## Abstract

Fucoidans are sulfated, fucose-rich marine polysaccharides primarily found in cell walls of brown seaweeds (macroalgae). Fucoidans are known to possess beneficial bioactivities depending on their structure and sulfation degree. Here, we report the first functional characterization and the first crystal structure of a prokaryotic sulfatase, PsFucS1, belonging to sulfatase subfamily S1_13, able to release sulfate from fucoidan oligosaccharides. PsFucS1 was identified in the genome of a *Pseudoalteromonas* sp. isolated from sea cucumber gut. PsFucS1 (57 kDa) is Ca^2+^ dependent and has an unusually high optimal temperature (68 °C) and thermostability. Further, the PsFucS1 displays a unique quaternary hexameric structure comprising a tight trimeric dimer complex. The structural data imply that this hexamer formation results from an uncommon interaction of each PsFucS1 monomer that is oriented perpendicular to the common dimer interface (~ 1500 Å^2^) that can be found in analogous sulfatases. The uncommon interaction involves interfacing (1246 Å^2^) through a bundle of α-helices in the N-terminal domain to form a trimeric ring structure. The high thermostability may be related to this unusual quaternary hexameric structure formation that is suggested to represent a novel protein thermostabilization mechanism.

## Introduction

Brown seaweeds (brown macroalgae) contain fucoidan polysaccharides as part of their cell wall. The glycosidic backbone of fucoidan mainly consists of L-fucose residues linked by either α-1,3 glycosidic bonds or by alternating α-1,3 and α-1,4 bonds. The latter is a typical pattern in *Fucus* species, e.g. *Fucus evanescens*^1^. In other species, such as *Turbinaria ornata* and *Sargassum mcclurei*, the fucoidans are galactofucans, and *S. mcclurei* fucoidan can even have up to a 1:1 ratio of fucose to galactose in the backbone^2^. Fucoidans have attracted attention due to their manifold beneficial bioactivities that depend on the fucoidan configuration including the sulfation degree^3–9^. In fucoidans from e.g. *F*. *evanescens* and *S. mcclurei* the sulfate groups are mainly found on C2 and/or C4 of the fucosyl moieties^1,2^ (in *S. mcclurei* fucoidan also on some of the galactosyl moieties^2^), while C3 sulfation is rare^1,2^. The complexity of fucoidans requires that the organisms feeding on these polysaccharides possess an array of specific degradative enzymes, both glycosyl hydrolases and sulfatases^10,11^. Insight into these enzymes is a prerequisite for solving the puzzle of how bacteria degrade macroalgal polysaccharides and notably provide an important foundation for developing targeted, selective fucoidan modification processes to study and enhance specific fucoidan bioactivities. A few endo-acting fucoidanases that specifically catalyze cleavage of α-1,4 fucosyl^12–14^ or α-1,3 linkages^15,16^, have been described, while two fucoglucuronomannan specific endo-fucoidanase lyases have also been identified^15^.

Sulfatases represent a large family of enzymes that catalyze the hydrolytic cleavage of sulfate ester groups on carbohydrates (EC 3.1.6.-sulfuric ester hydrolases; EC 3.10.1.-sulfamidases) and are divided into four classes based on homology, crystal structures, and catalytic mechanism^17^. Type I sulfatases (S1) include the vast majority of the currently characterized sulfatases^18,19^. To function, these enzymes require a post-translational modification of the conserved catalytic site cysteine or serine into a formylglycine, usually catalyzed by a formylglycine-generating enzyme^20,21^.

A few prokaryotic polysaccharide-specific type S1 sulfatases have been characterized from marine bacteria such as *Pseudoalteromonas carrageenovora*^22^, *Flammeovirga pacifica*^23^, *Pyrococcus furiosus*^24^ and *Thermotoga marina*^25^ with activity on agar, while carrageenan active sulfatases have been found in *Pseudoalteromonas atlantica*^26,27^, *Pseudoalteromonas* sp. PS47^18^ and *P. carrageenovora*^22,28^. Likewise, a few fucoidan active sulfatases have been reported^29–37^, although only two have been functionally characterized, namely SWF1 and SWF4 from the marine bacterium *Wenyingzhuangia fucanilytica* CZ1127T^36^. SWF1 and SWF4 show activity on fuco-oligosaccharides; SWF1 appears to be exo-acting^36^.

Here we report the discovery, expression, characterization and structural elucidation of the recombinant fucoidan active sulfatase PsFucS1. PsFucS1 was isolated from *Pseudoalteromonas* sp. from the gut of sea cucumbers. The data represent the first characterized and crystallized subfamily S1_13 sulfatase as well as the first crystal structure of a fucoidan active sulfatase.

## Results

### Identification of putative sulfatases in a sea cucumber gut bacterium

To identify new fucoidan active sulfatases, 97 strains of aerobic bacteria (MB01-97) were isolated from sea cucumber guts and selected on fucoidan agar plates. Fucoidan modifying ability was observed for 16 strains (Supplementary Fig. 1), three of which, MB47, MB87 and MB104, showed distinct sulfatase activity (Supplementary Fig. 2). The sulfatase activity was highest in strain MB47 (Supplementary Figs. 2 and 3), which by 16S ribosomal DNA comparisons was determined to be a *Pseudoalteromonas* sp., closely related to *Pseudoalteromonas shioyasakiensis* (Supplementary Table 1). The strain was genome sequenced, resulting in a 100% complete genome draft (VFBD00000000; Supplementary Table 2).

A range of carbohydrate-active enzymes (CAZymes) were predicted from the genome (Supplementary Table 3), including putative enzymes active on seaweed carbohydrates, e.g. carrageenan (GH16), agar (GH16) and alginate (PL6, PL7 and PL17). The many predicted CAZymes suggests that this marine bacterium can degrade several complex seaweed carbohydrates.

Seven putative sulfatases, containing the sulfatase domain signature IPR000917, were also identified in the genome (Supplementary Table 4). Five of these proteins, named PsSUL1 to 4 and PsFucS1 contained the conserved sulfatase domain (CXXXRXXXXXG). BLASTp comparisons using the SulfAtlas database (v1.2; http://abims.sb-roscoff.fr/sulfatlas)^17^, grouped these putative sulfatases into subfamilies S1_4 (PsSUL2) and S1_13 (PsFucS1, PsSUL1, PsSUL3-4; Supplementary Table 4). While the subfamily S1_4 contains several characterized sulfatases (EC 3.1.6.1), the specificity of S1_13 sulfatases is unknown. Multiple alignment of protein sequences (Supplementary Fig. 4) revealed that PsSUL2 is slightly related to ARS (identity 30%) (previously AtsA; accession number: P51691) from *Pseudomonas aeruginosa* PAO1, which is active on small sulfated compounds, including pNCS ^38,39^, while PsSUL1, 3, 4 and PsFucS1 seem more related to the agar sulfatase Ary423 from *F. pacifica* (identity 51, 47, 46 and 29% respectively; Accession Number: AKL72071.1)^23^.

### Functional characterization of the PsFucS1 sulfatase

The *Pseudoalteromonas* sp. MB47 sulfatases PsSUL1-4, PsFucS1 and the previously characterized sulfatases ARS and Ary423, used as benchmark sulfatases, were expressed in *Escherichia coli* and purified (Supplementary Fig. 5; Supplementary Table 5). ARS and PsFucS1 showed sulfatase activity, while the other sulfatases did not (Supplementary Fig. 6). Presumably, the heterologous expression in *E. coli* results in a mixture of unmodified and modified enzymes. Insufficient formylglycine formation by the heterologous formylglycine-generating enzyme in *E. coli* could therefore be the reason for the lack of activity of some of the sulfatases, while some of the ARS and PsFucS1 enzymes produced have been sufficiently transformed to result in measurable activity. All hitherto investigated enzymes in the S1 family of sulfatases require formylglycine modification for activity^18^. Hence, it is most likely that both modified and unmodified PsFucS1 molecules were present in the heterologously produced samples, and that a sufficient amount of PsFucS1 (and ARS) had been modified via the *E. coli* formylglycine-generating enzyme to elicit activity.

PsFucS1 exhibited activity between pH 6.0 to 7.0 with optimum at pH 6.5 and 68 °C (Supplementary Fig. 7). The kinetics analysis of PsFucS1 on pNCS showed that in the absence of NaCl, the apparent K_M_ was 0.95 mM, k_cat_ 1.5 min^−1^ and k_cat_/K_M_ was 1.6 mM min^−1^. In the presence of 125 mM NaCl, the apparent K_M_ was similar (0.88 mM) (Supplementary Fig. 8), but k_cat_ was reduced to 1.1 min^−1^ and k_cat_/K_M_ to 1.3 mM min^−1^.

The thermostability analysis verified an extraordinarily high thermal stability of the PsFucS1 at 68 °C with a slightly negative effect of the presence of NaCl. Hence, when pre-incubated without NaCl, k_D_ of the enzyme was 9.5 × 10^–4^ min^−1^ at 68 °C (half-life 12.2 h); but when incubated with 125 mM NaCl the k_D_ of the enzyme was higher, 13.4 × 10^−4^ min^−1^ (half-life 8.6 h) (Supplementary Figs. 9 and 10).

### Crystal structure of PsFucS1

Although about forty crystal structures of sulfatases have been characterized to date, none are from the sulfatase subfamily S1_13 like PsFucS1 as defined by the SulfAtlas database^17^. The X-ray structure of PsFucS1 was solved by molecular replacement at 2.50 Å resolution (PDB entry 7AJ0), using the human N-acetylgalactosamine-6-sulfatase (GALNS; NM_000512.4; 4FDI; S1_5) as starting model. The crystals belonged to space group P2_1_2_1_2_1_ with six molecules in the asymmetric unit. The RMSD values of Cα atoms between the six copies were low, ranging from 0.10 to 0.15 Å, and the monomers can therefore be considered as identical (detailed data statistics are summarized in Supplementary Table 6).

**Figure 1:**
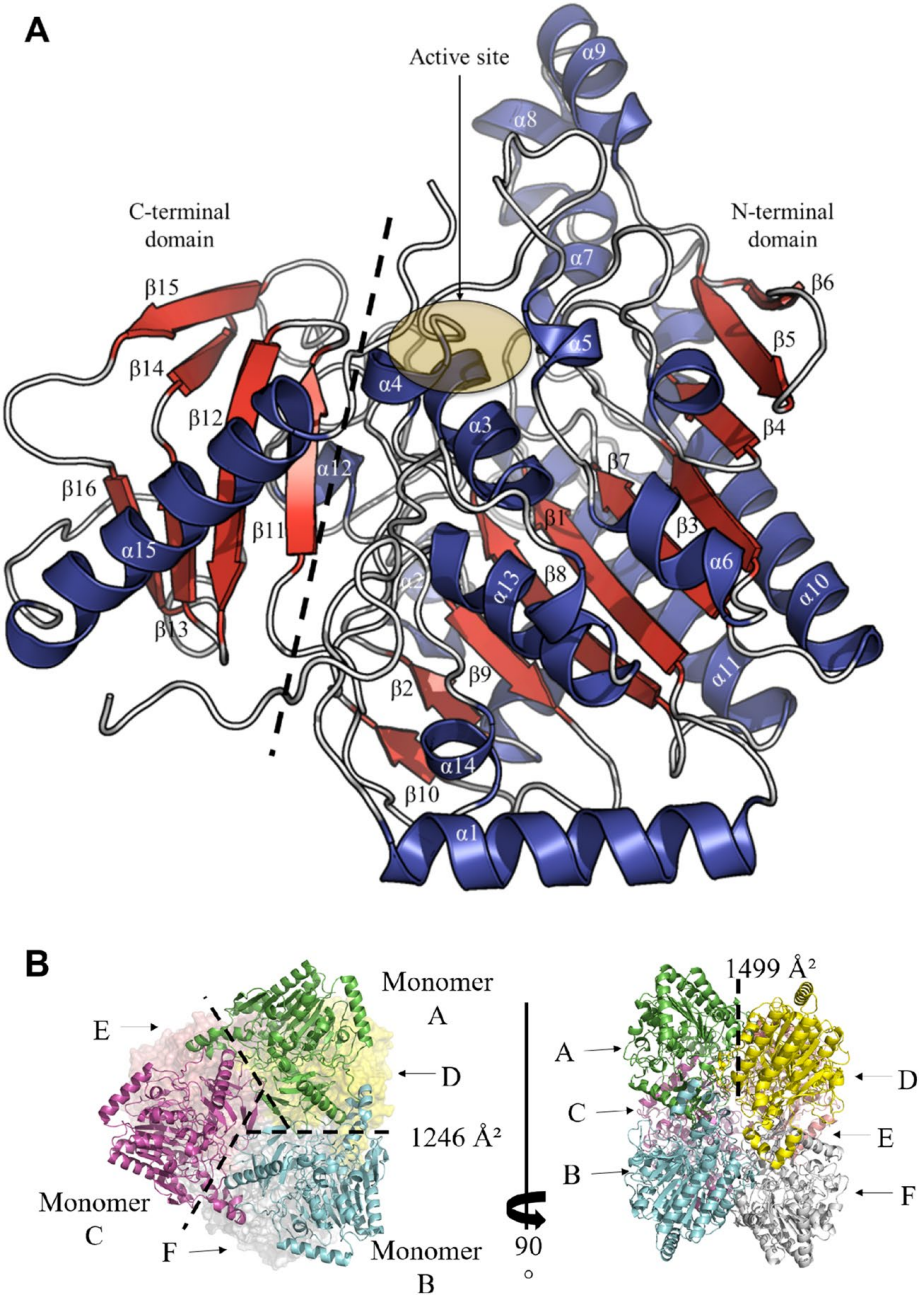
PsFucS1 fold and oligomeric state. (**A**) Cartoon representation of the PsFucS1 structure showing the domain organization and secondary structure elements. The N-terminal domain (Asn21-Leu419: α1-α14 helices and β1–β10 strands) and the C-terminal domain (Val430-Gly521: α15 helix and β11–β16 strands) are separated by a dashed line. The active site position is circled in yellow. Secondary structure elements are colored in red and blue for β-strands and α-helices, respectively. (**B**) Cartoon representation of the PsFucS1 hexamer (trimer of dimers) in the asymmetric unit. Monomers A, B, C, D, E and F are colored in green, cyan, purple, yellow, pink and white, respectively.

The overall structure (Fig. 1A) revealed that each PsFucS1 monomer is organized in a catalytic N-terminal domain (Asn21-Leu419) region and a C-terminal domain (Val430-Gly521) typical of the formylglycine dependent sulfatase fold. The quaternary organization of the molecules in the asymmetric unit of the crystal can be described as a trimer of dimers (Fig. 1B), the dimer being similar to the closest homologous sulfatase 4FDI, while the trimer interface appears unique to PsFucS1.

To further decipher the oligomeric state of PsFucS1 in solution, the enzyme was analyzed by size-exclusion chromatography. A single peak (Supplementary Fig. 11) with an estimated apparent molecular mass of approximately 291 kDa was observed, corroborating a hexameric structure of PsFucS1 in solution, the theoretical molecular mass of which is calculated to be 345.8 kDa (and 172.4 kDa for a trimer).

The common dimer interface, already observed in numerous sulfatases, is approximately 1500 Å^2^ (predicted by PISA, Fig. 1B) and involves 48 residues. Interfacing residues are mainly found in loops and very few are found in secondary structure elements. Surprisingly, interfacing residues are not that conserved within the sulfatase subfamily S1_13 (approximately 25%) and half of them are not conserved (present in less than 5% of the sequences). However, among the 48 interfacing residues, Gly80, Thr103, Arg363 and Asp482 residues are highly conserved (> 90%) and potentially play a key role in dimerization. The Gly80 residues of both monomers are face-to-face to avoid steric clashes. The Arg363 residue, stabilized by O-Thr103, Oγ-Ser104 and Oδ1-Asn381 atoms of the same monomer, is hydrogen-bonded to the O-Ala84 atom of the other monomer. The Asp482 residue participates in the construction of a π-turn structure and the formation of hydrogen bonds with N-Glu485, N-Met486 and N-Ala487 atoms. The π-turn located in the loop connecting the β16-strand to the α15-helix surely allows the α15-helix to cap β-strands of the C-terminal domain.

The trimer interface of each monomer is approximately 1246 Å^2^ (predicted by PISA, Fig. 1B) and involves 76 residues. The crystal structure of PsFucS1 is the first representative of the sulfatase subfamily S1_13, and interfacing residues, found both in loops and secondary structure elements in the trimer are poorly conserved (approximately 13%). This atypical interface is created through a bundle of α-helices (α7, α8 and α9) found in the N-terminal domain and composed of residues with very low conservation. Like the classic dimer interface, only four residues (His281, Lys354, Gly355 and Pro474) are highly conserved (> 90%) in the interface between each monomer forming the trimer.

### Active site of PsFucS1

**Figure 2:**
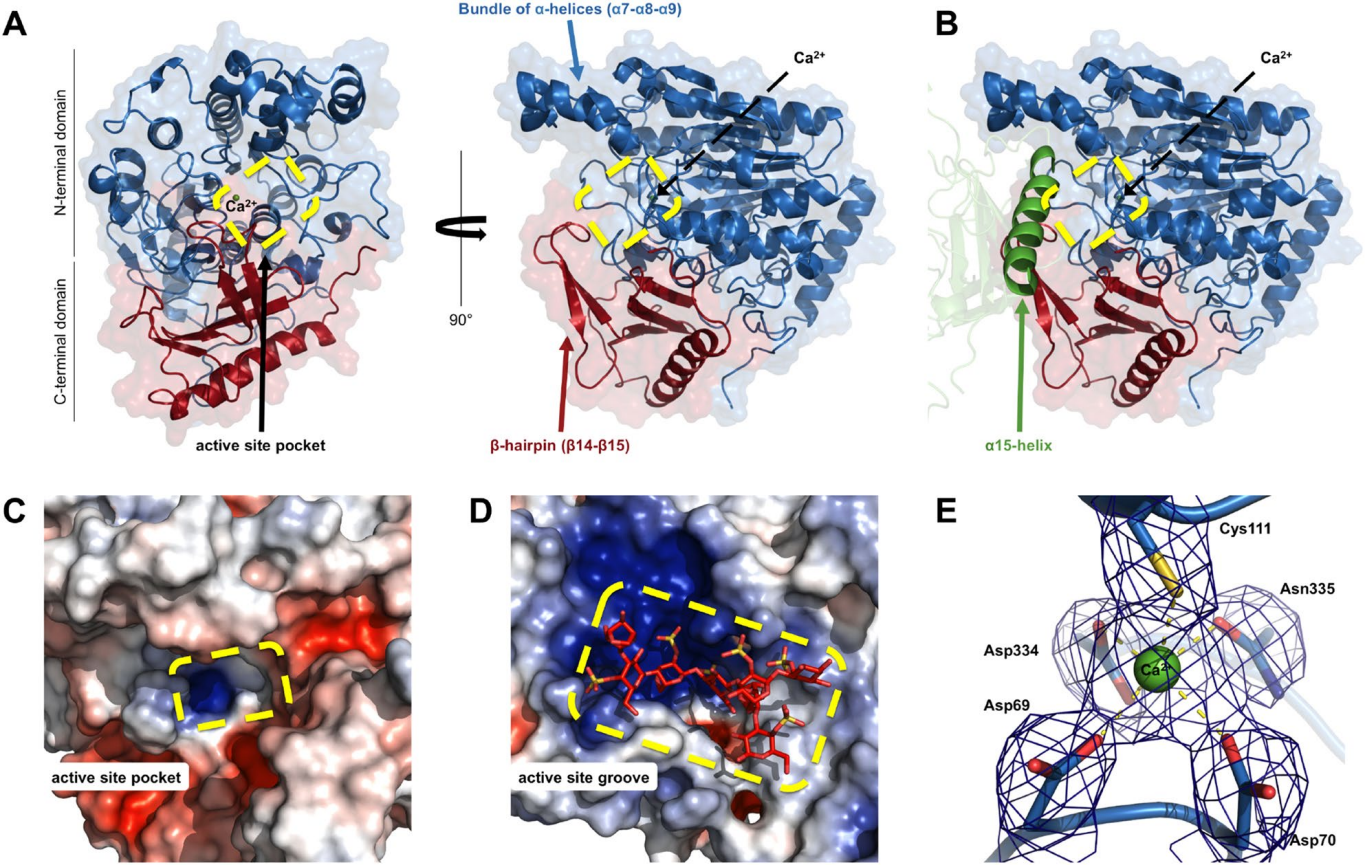
PsFucS1 active site topology. (**A**,**B**) Cartoon representation of PsFucS1. The N-terminal domain and the C-terminal domain are colored in blue and red, respectively. The active site pocket is circled in yellow. Structural elements delineating the protein active site are indicated by arrows. In (**B**) Part of the interfacing neighboring monomer is indicated in green including the α15 helix that partially covers the active site. (**C**) PsFucS1 crystal structure in electrostatic representation. The active site pocket is circled in yellow. (**D**) Electrostatic representation of the endo-4S-ι-carrageenan sulfatase from *Pseudoalteromonas* sp. PS47 (6B0J) in complex with κ-ι-κ-neocarrahexaose shown for comparison^18^. The active site groove is circled in yellow. Electrostatic potential is expressed as a spectrum ranging from − 5 kT e^−1^ (red) to + 5 kT e^−1^ (blue) and was calculated in APBS^77^. (**E**) Electron density around the calcium ion of PsFucS1. The map shown is a σA-weighted 2mFo-DFc map contoured at 1.2σ (0.67e^−^ Å^−3^). Asp69, Asp70, Cys111, Asp334 and Asn335 residues are shown as sticks. The calcium atom is shown as a green sphere.

The active site of PsFucS1 (Fig. 2A) is delineated on one side by the presence of an extended β-hairpin loop and on the other side by a bundle of three α-helices, displaying a pocket topology. It is interesting to notice that the α15-helix coming from the other monomer (Fig. 2B) covers the active site. Mapping electrostatic potential onto the surface of PsFucS1 highlights a positively charged pocket (Fig. 2C), perfectly suited for the recognition of a sulfate group. The positive charge at the active site is similar to that of a 4S-ι-carrageenan active endo-sulfatase (6B0J; Fig. 2D), crystallized with a substrate molecule. The active site of sulfatases normally encompasses the catalytic nucleophile formylglycine residue but the Fo-Fc difference density of the X-ray structure of PsFucS1 showed no evidence of significant maturation of the catalytic Cys111, but undoubtedly showed the unmodified cysteine. This is common in crystal structures of sulfatases that are heterologously produced in the absence of the native formylglycine-generating enzyme, since the *E. coli* maturation machinery is often unable to post-translationally modify the sulfatase to a substantial extent^18^. If modified formylglycine residues are present in the crystal, they appear to be the minor fraction and are averaged out by the crystal structure.

The catalytic residue Cys111 together with Asp69, Asp70, Asp334, and Asn335 coordinates a calcium ion (Fig. 2E) required for the binding and activation of the sulfate group of the substrate, which is highly conserved among S1 sulfatases. Analysis of the effect of presence of divalent cations on PsFucS1 activity showed profound activity retarding effects of the heavy metals Ni^2+^, Zn^2+^, Cu^2+^, and Fe^2+^, while addition of Mg^2+^, Mn^2+^ and Ca^2+^ produced increased activity of 127, 131 and 608%, respectively (Table 1), verifying that Ca^2+^ is indeed required for optimal activity of PsFucS1.

**Table 1:** Effect of divalent cations on PsFucS1 activity.

Divalent cations	Relative activity (%)^1^			
	0 mM	2 mM	5 mM	10 mM
Ca^2+^	100^d^	258^c^ ± 16	452^b^ ± 16	608^a^ ± 4
Mg^2+^	100^a^	110^a^ ± 12	124^a^ ± 12	127^a^ ± 8
Mn^2+^	100^b^	131^a^ ± 9	nd	nd
Ni^2+^	100^a^	16^b^ ± 1	15^b^ ± 1	nd
Zn^2+^	100^a^	6^b^ ± 3	nd	nd
Cu^2+^	100^a^	35^b^ ± 6	nd	nd
Fe^2+^	100^a^	8^b^ ± 1	nd	nd

*nd* not determined.

^1^Data are given as averages of duplicate determinations in each row in this Table. Different roman superscript letters a–c indicate statistically different values at *p* < 0.05 for different addition levels of each cation.

**Figure 3:**
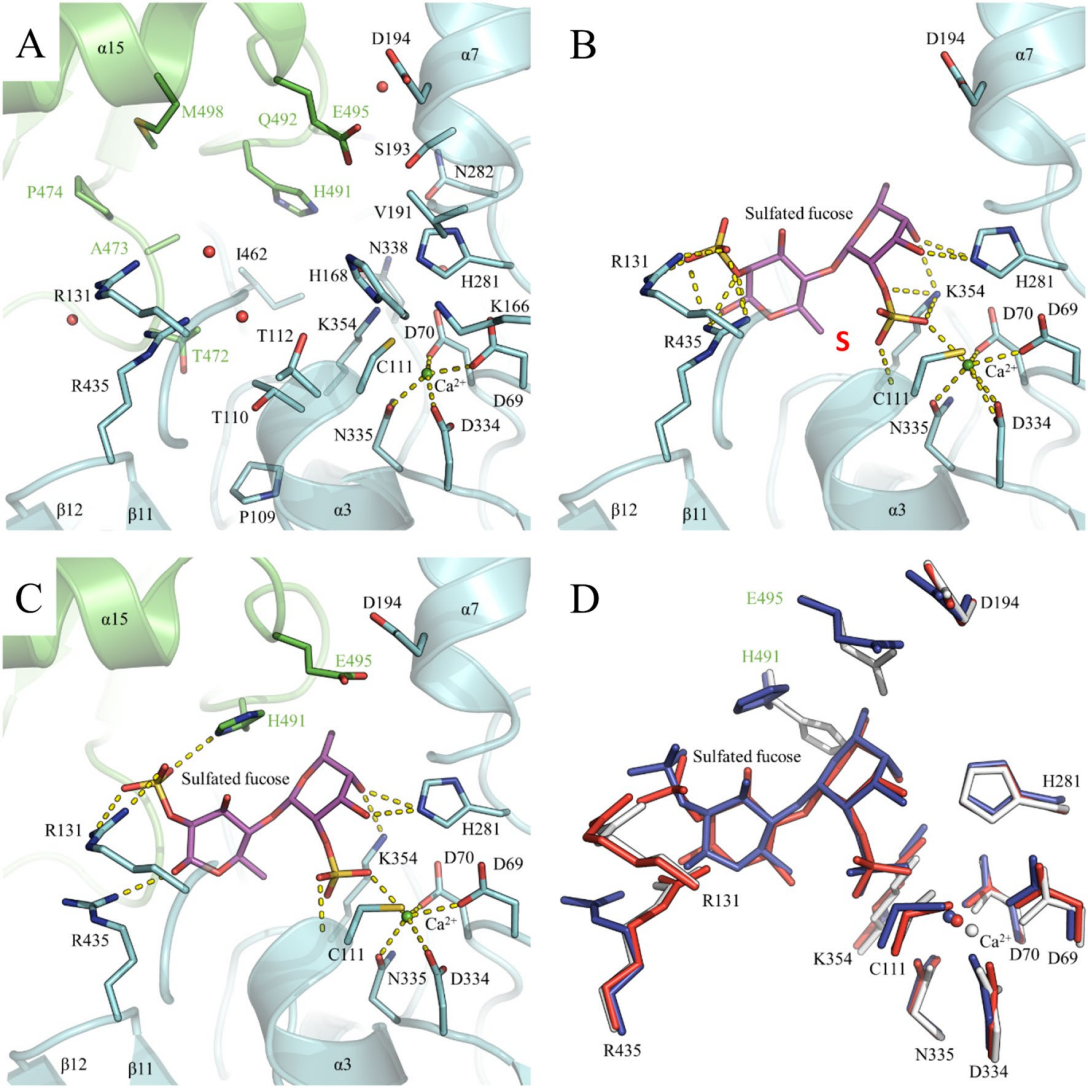
Docking results of sugar-sulfatase complexes and PsFucS1 activity on fucoidan. (**A**) Active site pocket of PsFucS1 X-ray crystal structure. (**B**) Cartoon representation of C2 sulfated fucobiose-PsFucS1 complex obtained by docking. The red S indicates the position of the sulfate binding site as defined by Hettle et al. 2018^18^. (**C**) Zoomed-in view of PsFucS1 active site with docked disaccharide at the dimer interface. (**A**–**C**) Monomers A and B are colored in green and cyan, respectively. Residues constituting the binding pocket are pointed as sticks and those from monomer A are labeled in green. Secondary structure elements are shown as cartoon. The calcium atom and water molecules are shown as green and red spheres, respectively. The bound disaccharide into the active site pocket is shown as purple sticks. H bonds between the ligand and protein are yellow dotted lines. (**D**) Superposition of the PsFucS1 X-ray crystal structure (white) to the monomer docked model (red) and the dimer docked model (blue).

The subsite nomenclature for carbohydrate sulfatases is such that the invariant sulfate binding site is denoted the S site. The sulfate of this site is appended to the 0 subsite sugar unit. Sugar subsites then increase in number (i.e. + 1, + 2) as the sugar moves toward the reducing end (free O1) and decreases in number as the sugar chain moves towards the non-reducing end (i.e. − 1, − 2)^18^. Molecular docking of C2 sulfated fucobiose into the active site (Fig. 3) allowed to position L-fucose units in subsites 0 and + 1^18^ by replacing water molecules of the PsFucS1 crystal structure (Fig. 3A), and with the sulfate group correctly positioned at the S-site (defined in Hettle et al. 2018^18^; Fig. 3B).

The interactions observed in the docked ligand-enzyme structure (Fig. 3B–D) highlight the essential residues that possibly determine the observed substrate specificity. The best docking score was obtained in complex with a C2-sulfated fucobiose molecule as ligand. The C2-sulfate of fucose bound in subsite 0 is ideally positioned for catalysis, and the C2-sulfate of the adjacent fucose unit, in subsite + 1, is tightly bound by two arginine residues (namely Arg131 and Arg435, Fig. 3B,C) that are π-stacked to another. In this configuration the fucose unit at subsite 0 is further recognized by His281 (hydrogen bonded to O3 and O4) and Lys354 (hydrogen bonded to O3). Interestingly, one loop of a neighboring monomer (within the dimer) comes to lie right next to the + 1 site, possibly participating in the recognition of the sulfated-fucose oligosaccharides. Notably, this loop contains two residues, His491 and Glu495 that could be crucial for substrate specificity: His491 participates in binding of the fucose in subsite + 1, while the presence of Glu495 would sterically interfere with a sulfate at C3 of this fucose unit (Fig. 3C).

### Sulfatase activity of PsFucS1 on fucoidan oligosaccharides

Examination of the sulfatase activity of PsFucS1 on fucoidans from *S. mcclurei* and *F. evanescens* revealed that PsFucS1 did not release detectable amounts of sulfate from native fucoidans (Table 2), but showed that the enzyme released sulfate from fucoidan oligosaccharides (Table 2) prepared by endo-fucoidanase treatment^12,15,40^. Of the total sulfate in the respective substrates, PsFucS1 released of 0.6% sulfate from *F. evanescens* fucoidan and 1.0 or 5.5% respectively from *S. mcclurei* fucoidan, dependent on the fucoidanase used. PsFucS1 activity on fucoidan oligosaccharides was, as expected, higher at 68 °C than 35 °C (Table 2).

**Table 2:** Activity of PsFucS1 in combination with or without different fucoidanases on different fucoidan substrates at two different temperatures.

Fucoidan origin	Total sulfate content (quantified as %SO_4_^2−^)	Reaction temp. (°C)	%SO_4_^2−^ released by PsFucS1^1^			
			**–**	FdlA	FdlB	FcnAΔ229
*Fucus evanescens*	30.3 ± 1.9	35	0^b,x^	0.2^a,y^ ± 0.0	0.1^a,y^ ± 0.0	0.2^a,y^ ± 0.0
		68	0^b,x^	0.6^a,x^ ± 0.2	0.6^a,x^ ± 0.0	0.6^a,x^ ± 0.1
*Sargassum mcclurei*	32.9 ± 2.3	35	0^c,x^	1.3^a,y^ ± 0.0	1.3^a,y^ ± 0.0	0.7^b,y^ ± 0.0
		68	0^c,x^	5.5^a,x^ ± 0.0	5.5^a,x^ ± 0.0	1.0^b,x^ ± 0.0

– no fucoidanase.

^1^Sulfate-release data (%SO_4_^2−^ released) are given as averages of duplicate assays (data are relative to the theoretical maximum release); different roman superscript letters a–c indicate significantly different values (*p* < 0.05) in each row, i.e. in the PsFucS1 reactions together with the different fucoidanases (FdlA, FdlB, and FcnAΔ229), respectively, and different roman superscript letters x–y indicate significantly different values (*p* < 0.05) when comparing the two reaction temperatures for each type of fucoidan reaction.

## Discussion

Considering its mesophilic marine bacterium origin, PsFucS1 had a surprisingly high thermal reaction optimum at 68 °C (at pH 6.5) and an unusually high thermostability. Three thermostable sulfatases have been described to date, the agar sulfatase from *T. maritima* with optimal temperature at 80 °C^25^, Atsa from *P. aeruginosa* with optimal temperature of 57 °C^38^ and Ary423 from *F. pacifica*, with optimal activity at 40 °C, but with a wide temperature range of 30–70 °C^23^. Ary423 retained more than 40% activity at 60 °C after 12 hours^23^, but the stability of PsFucS1 was even higher with a half-life at 68 °C of 12.2 h.

Interestingly, PsFucS1 formed hexamers in the crystal as well as in solution. Higher oligomerization has frequently been observed as a strategy to attain thermostability^41^, suggesting that the thermostability of PsFucS1 might be obtained through hexamerization. It is noteworthy that a recent report also links higher oligomerization states for cold adaptation of an enzyme^42^. While several oligomerization states, ranging from monomer to tetramer and even an octamer (1auk, family S1_1), have been observed for different S1 sulfatases so far, the hexameric organization of PsFucS1 observed here is unique. Among S1_13 sequences the crucial regions leading to the trimerization are not conserved. It is thus highly probable that not all S1_13 sulfatases occur as hexamers. The enzyme was Ca^2+^ dependent, in accord with the structural protein analysis that clearly indicated a negatively charged calcium cavity near the active site. Despite being of marine origin, PsFucS1 was both more active and more stable without NaCl than in the presence of NaCl with a 1.3 fold higher k_cat_ without NaCl, but a similar K_M_ value. Together with the thermal stability data these results might suggest that conditions with warmer temperatures and lower salt concentrations than found in the Vietnamese sea, could be the true origin of the PsFucS1 *Pseudoalteromonas* sp. MB47 bacterium.

PsFucS1 presumably removes sulfate from short fuco-oligosaccharides in an exo-acting manner. Only a small amount of sulfate was released as expected, largely due to the lack of complete degradation of the fucoidan by the fucoidanases, and due to the differentially positioned sulfate groups. Indeed, this hypothesis can also be inferred from the fucobiose-PsFucS1 complex that was obtained by molecular docking, where the complex with a C2-sulfated fucobiose molecule as ligand gave the best score. Moreover, the formylglycine post-translational modification might, as suggested above, not be fully obtained during the heterologous expression in *E. coli*, likely resulting in the lower apparent activity of the sulfatase. This interpretation is supported by the crystal structure, where no formylglycine was evident in the active site of the crystallized enzymes.

In summary we report the biochemical characterization and crystal structure of a hexameric sulfatase that releases sulfate from fucoidan oligosaccharides. Moreover, the PsFucS1 has a promising advantage of high thermostability, which may be attractive for application in production of partially desulfated fucoidan oligosaccharides in the future. Indeed, many bioactivities have been shown for fucoidans and fucoidan oligosaccharides^3,5,43^, while the exact underlying chemical structures responsible for the various bioactivities remain elusive. By the use of fucoidan sulfatases, including PsFucS1, the position-function relationship of sulfate groups on fucoidan for the bioactivity could potentially be elucidated.

## Methods

### Substrates

*p*-nitro catechol sulfate dipotassium salt (pNCS) was from Sigma-Aldrich (Steinheim, Germany). 5-Bromo-4-chloro-3-indolyl sulfate potassium salt (X-SO_4_) was from Carbosynth Ltd (Compton, UK). Fucoidans from *S. mcclurei* and *T. ornata* were obtained as described previously^2^. The F3 fraction of *S. mcclurei* fucoidan was obtained by anion exchange chromatography^2,44^ using DEAE-macroprep resin (Bio-Rad, Hercules, CA, USA). Crude fucoidans from *F. evanescens* were extracted using an enzyme-assisted method and further fractionated to obtain the F2 fraction by anion exchange chromatography using a DEAE-macroprep resin (as above), and characterized as previously described^45^.

### Isolation of fucoidan-utilizing marine bacteria

Sea cucumbers were sampled at Hon Tre, Nha Trang Bay, Vietnam (12.23100 N, 109.24203 E). The sea cucumbers were stored on ice and transported to the laboratory within an hour of collection. After the surface of each of the sea cucumbers was sterilized with 70% ethanol, the ventral surface was dissected with a sterile scalpel and the digestive tract was removed and its contents were carefully squeezed into a sterile tube. All gut content and sediment samples were carefully mixed with sterile sea water and suspensions were spread on solid fucoidan MB media (F-MB) containing 15 g L^−1^ agar, 5 g L^−1^ Difco bactopeptone, 2 g L^−1^ Difco yeast extract, 1 g L^−1^ crude fucoidan from *S. mcclurei*, 0.2 g L^−1^ K_2_HPO_4_, and 0.05 g L^−1^ of MgSO_4_. Sampled seawater was used for media preparation (pH 7.5–7.8). After growth at 28–30 °C for 1–7 days, bacterial colonies were streaked on solid MB medium to obtain pure cultures.

### Screening of marine bacteria for enzymatic activity

The bacterial isolates were screened for fucoidan-modifying enzymes by the fucoidan agar plate cetavlon method^46^. In brief, isolates were cultivated for 3 days at 28 °C on fucoidan agar medium containing 0.5% (w/v) *S. mcclurei* or *T. ornata* fucoidan. Bacterial cells were removed from the agar plate surface and a 1% aqueous solution of the cationic hexadecyltrimethylammonium bromide salt (cetavlon; Sigma-Aldrich, Steinheim, Germany) was added to each plate. After incubation for 30 min at 25 °C, the agar plates were thoroughly washed with water. Presence of the cationic cetavlon salt forms a water-insoluble white precipitate in the presence of fucoidan: hence transparent zones indicate that the bacteria secrete enzymes with fucoidan-modifying activity since enzymatically degraded fucoidan has a lowered charge density and will not precipitate with cetavlon. Sulfatase activity was detected using MB solid media supplemented with 100 μg ml^−1^ X-SO_4_. Enzymatic activity was detected by blue color formation around the colonies^38^.

### Strain identification by 16S ribosomal RNA analysis

Bacterial cells from 5 ml overnight culture in MB liquid media were collected by centrifugation and genomic DNA of selected strains was isolated by DNeasy Blood and Tissue DNA kit (Qiagen, Hilden, Germany), following the manufacturer’s recommendations. The 16S rRNA gene fragment was amplified using Phusion High-Fidelity DNA Polymerase (New England Biolabs, Ipswich, MA, USA) with the universal bacterial primers (533F: 5’-GTGCCAGCAGCCGCGGTAA3’ and 1392R: 5’-GGTTACCTTGTTACGACTT-3’), blunt-end cloned into pJET1.2 vector using CloneJET PCR Cloning Kit (ThermoFisher Scientific, Waltham, MA, USA) and propagated in the *E. coli* DH5α strain (Invitrogen Life Technologies, Thermo Fisher Scientific, Waltham, MA, USA). After plasmid preparation, inserts were sequenced at Macrogen Inc. (Seoul, Korea) using the Sanger capillary sequencing method. Sequences were aligned against 16S ribosomal RNA entries of NCBI RefSeq database to identify the closest taxonomic bacterial identity.

### Genome sequencing of and sequence assembly

Genomic DNA of the *Pseudoalteromonas* sp. MB47 strain was isolated as described for the 16S ribosomal analysis. The genomic DNA was submitted to Macrogen Inc. (Seoul, Korea) for Illumina TruSeq DNA PCR-free shotgun library preparation and sequencing using Illumina Miseq (300 bp paired-end). Residual sequencing adapters from resulting reads were removed with Cutadapt^47^ and BBDuk^48^. After quality filtering, draft genomes were assembled with Ray using a number of different k-mer lengths^49^. Best assemblies were selected based on the assessment of the constructed deBrujin graphs and quality of the resulting contigs. Reads were mapped back to contigs using the Bowtie2 algorithm^50^. To further clean assemblies abundance/GC binning was performed with MetaBat^51^. The completeness of the resulting draft genome was evaluated using Checkm^52^. Draft genome annotation and CAZymes identification protein-coding features and ribosomal RNA features were predicted with Prodigal and barrnap as part of the prokka annotation pipeline. Additionally, all predicted protein sequences were subjected to annotation using the Interproscan pipeline (v5.27-66.0), using all available databases and analyses. Taxonomy was investigated by aligning all predicted protein sequences against the NR database. Analysis of selected phylogenetic protein markers was done using AMPHORA2^53^ and analysis of identified sequences encoding 16S rRNA genes. dbCAN models were used to predict CAZymes^54^.

### Identification of sulfatases in the *Pseudoalteromonas* sp. genome and sequence alignment

Protein-coding features in the *Pseudoalteromonas* sp. MB47 genome were predicted with Prodigal (used as part of the prokka annotation pipeline)^55^ and subjected to annotation using Interproscan pipeline^56^, v5.27-66.0), using all available databases and analyses. All signatures corresponding to sulfatase families and domains have been retrieved from the InterPro database^57^ and used for extraction of sulfatase protein sequences. Signal peptides were predicted using SignalP 4.0^58^. SulfAtlas (http://abims.sb-roscoff.fr/sulfatlas) was used to identify the sulfatase families and subfamilies.

Multiple sequence alignments were performed using ClustalW^59^ via the NPS@ server (https://npsa-prabi.ibcp.fr/)^60^. Sequences included previously characterized polysaccharide-active sulfatases as well as the human GALNS sequence. The alignment was rendered using ESPript 3.0^61^.

### Cloning, expression and purification of PsFucS1

#### Enzymes and gene constructs

Constructs containing the genes encoding the sulfatases were designed without the predicted N-terminal signal peptide but with an N-terminal 6xHis tag. The synthetic codon-optimized genes (optimized for *E. coli* expression), all devoid of their original signal peptide, were synthesized by GenScript (Piscataway, NJ, USA) and delivered inserted into the pET-45b(+) vector between the KpnI and PacI restriction sites. The *E. coli* strain DH5α (Invitrogen, Waltham, MA, USA), was used as plasmid propagation host.

#### Production of recombinant sulfatases and fucoidanases

Expression of the sulfatase and endo-fucoidanase FcnAΔ229 was performed in *E. coli* BL21 (DE3; Invitrogen, Waltham, MA, USA) harbouring the Pch2 (pGro7) plasmid, as previously described^40^, and expression of FdlA, FdlB was performed in *E. coli* C41 (DE3) as previously described^40^. The enzymes were desalted using a PD10 desalting column (GE Healthcare, Chicago, IL, USA) and the protein content was determined by Bradford assay^62^ with bovine serum albumin as standard.

#### SDS–PAGE and western blot analysis

The homogeneity and molecular weight of the recombinantly expressed proteins were estimated by sodium dodecyl sulfate–polyacrylamide gel electrophoresis (SDS–PAGE) in 12% acrylamide gels, according to the Laemmli protocol^63^ and western blot analysis using poly-histidine antibodies as previously described^40^. 7.5 µl *E. coli* culture, 40 µg crude protein and 5 µg purified protein was loaded on the gels. The Protein Plus molecular weight marker (Bio-Rad Laboratories, Hercules, CA, USA) was used as standard.

### Enzyme activity assays

#### X-SO_4_ plate screening

Whole *E. coli* cells harboring the different sulfatase gene-constructs were tested for sulfatase activity by streaking the cells onto X-SO_4_ agar plates (15 g l^−1^ agar, 20 mM Tris–HCl, 125 mM NaCl, pH 6.5 and 100 µg ml^−1^ X-SO_4_). Crude *E. coli* cell lysates containing recombinant sulfatases were tested on the X-SO_4_ agar plates by punching a hole in the plates and adding 50 µl of the cell lysate into the hole. All plates were incubated at 37 °C for 30 min to 24 h.

#### pNCS sulfatase assay

Sulfatase activity was measured by incubating enzyme samples (0.5 mg ml^−1^ sulfatase) in a reaction mixture of 40 mM Tris–HCl pH 6.5, 10 mM CaCl_2_, 125 mM NaCl, and 2.5 mM pNCS at 37 °C or 68 °C. The reaction was stopped by addition of NaOH (final concentration 0.8 M NaOH) before recording absorbance at 515 nm. One unit of sulfatase activity was defined as the amount of enzyme capable of hydrolyzing 1 µmol of pNCS per minute at the assay conditions. Kinetics experiments were performed with different pNCS concentrations (0.5, 2.5 and 4 mM) at pH 6.5, 68 °C and K_M_ and k_cat_ values of the sulfatase were calculated according to Michaelis–Menten kinetics^64^.

The influence of cations on PsFucS1 sulfatase activity was determined after pre-incubation with 0–10 mM CaCl_2_, MgCl_2_, MnCl_2_, NiCl_2_, CuCl_2_, ZnCl_2_, or FeCl_2_ for 5 min followed by assay on 2.5 mM pNCS at 37 °C as described above. After the calcium dependence was discovered 10 mM CaCl_2_ was included in all assays.

Effect of pH and temperature on sulfatase activity was assessed in a statistical factorial design (CCF) using two overlapping buffer systems ranging from pH 5.0 to 8.0 (200 mM acetate buffer pH 5.0 and 200 mM Tris–HCl buffer, center point pH 6.5) and reaction temperatures from 60 to 75 °C, with 67.5 °C as center point.

Thermostability of recombinant PsFucS1 was evaluated by measuring the residual enzyme activity after pre-incubation of the enzyme in 40 mM Tris–HCl, 10 mM CaCl_2_, pH 6.5 at 68 °C with various levels of NaCl 0–125 mM for up to 12 h. First-order rate constants of the thermal denaturation (K_D_) and half-life at 68 °C was calculated from the activity decay (Supplementary Figs. 9 and 10).

### Fucoidan desulfation

Desulfation of fucoidans was measured after fucoidan hydrolysis catalysed by different fucoidanases in reaction mixtures containing 0.5 µg µl^−1^ fucoidanase (FcnA, FdlA and FdlB) in 20 mM Tris–HCl buffer pH 7.4, 250 mM NaCl, 10 mM CaCl_2_ and 1% (w/v) fucoidans from either *S. mcclurei* or *F. evanescens* at 35 °C and 68 °C for 24 h. The PsFucS1 (1 mg ml^−1^) was then added to the reaction resulting in the following reaction mixture: 125 mM NaCl, 10 mM CaCl_2_, 60 mM Tris–HCl pH 6.5, 0.5% (w/v) fucoidan. Desulfation data were compared by one-way analysis of variance (ANOVA) using Tukey–Kramer’s Significant Differences at *p* ≤ 0.05 using JMP 15 Pro (SAS Institute Inc., Cary, NC, USA).

### Sulfate detection by ion-exchange chromatography

The sulfate content of fucoidans substrates as well as sulfate release after sulfatase treatment was analyzed by high performance anion exchange chromatography (HPAEC) using a Dionex ICS-5000 chromatography system equipped with a GP40 gradient pump and an ED40 electrochemical detector. The ions were separated on an HC-AS11 anion-exchange column (4 × 250 mm; Dionex) with accompanying HC-AG11 guard column (4 × 50 mm; Dionex). Elution was performed with 35 mM NaOH using an isocratic flow rate of 1.5 ml min^−1^. Background signal and noise originating from the eluent was reduced using an anion self-regenerating suppressor (AERS-500, Dionex) with a current of 180 mA. Sulfate concentration was deduced from the signal intensity and calculated from a standard sulfate calibration curve using Na_2_SO_4_. The release of sulfate was related to the amount of sulfate present in the polysaccharide substrate, 33 ± 3% in *S. mcclurei*^2^ and 30 ± 2% in *F. evanescens*^45^.

### Crystallization and structure determination

PsFucS1 crystals were grown in a hanging drop set up at 20 °C using 2 µl protein solution (6 mg ml^−1^) and 1 µl mother liquor solution containing 0.1 M sodium acetate pH 4.6, 0.1 M sodium chloride and 20% (w/v) PEG 3350. Crystals were flash-frozen and stored in mother liquor supplemented with 14% (v/v) glycerol prior to diffraction experiments. Diffraction data were collected on the PROXIMA-2A beamline at the SOLEIL Synchrotron. Data were processed using XDS^65^ and scaled and merged with Aimless^66^ in CCP4^67^. An initial homology model was generated from the SWISS-MODEL server^68^ based on the N-acetylgalactosamine-6-sulfatase X-ray structure from human (4fdi)^69^, with which PsFuc1 shares 25% sequence identity. Only residues with a predicted local similarity to target > 0.6 were kept for molecular replacement. The PsFucS1 crystal structure was then solved by molecular replacement using phenix.phaser^70^. Density modification and model building was carried out with phenix.autobuild^71^. Refinement was carried out using phenix.refine^72^ interspersed with manual model revisions using the program Coot^73^. All statistics are summarized in Table S6.

### Analytical size-exclusion chromatography (SEC)

Analytical SEC was performed using a HiLoad 16/600 Superdex 200 pg column connected to an ÄKTA-Purifier at room temperature. The column was calibrated with molecular mass standards ranging from 13.7 to 440 kDa. The molar mass of each protein and their elution volume are as follows: Ferritin (440 kDa—58.08 ml); Aldolase (158 kDa—69.71 ml); Ovalbumin (44 kDa—83.31 ml); Carbonic anhydrase (29 kDa—91.10 ml); Ribonuclease A (13.7 kDa—99.31 ml). 200 µl of His-tagged PsFucS1 (6 mg ml^−1^) were loaded on the column equilibrated with 50 mM Tris–HCl buffer pH 8.0 and 100 mM NaCl at a flow rate of 0.5 ml min^−1^. The absorbance of the eluted fractions was recorded at 280 nm.

### Molecular docking with a sulfated fucose molecule

Molecular docking was performed on a single PsFucS1 monomer. Only protein atoms were kept (hydrogen, water molecules, and chloride atoms were removed). The initial coordinates of a C2-sulfated fucobiose molecule was generated from the smiles string using phenix.elbow^74^. Then, AutoDock vina^75^ was used to obtain a docking model of the PsFucS1/C2-sulfated fucobiose complex. Before docking, the side chains of residues in the active site pocket were set as flexible. Docking was done over a search space of 20 × 20 × 20 Å that covers the entire active site pocket. The calculation yielded 10 possible models, of which the one with the highest ranking was selected as the most likely. Finally, the PsFucS1 structure in complex with the C2-sulfated fucobiose was energy minimized using UCSF Chimera^76^ with standard steepest descent and conjugate gradient steps. In addition, after the first energy minimization, the C2-sulfated fucobiose molecule binding site at the dimer interface was generated by superposition of the structures followed by energy minimization of the dimer in complex with the substrate using UCSF Chimera^76^.

## Supplementary Information

Supplementary Information.

## Data Availability

The crystal structure presented has been deposited with PDB entry 7AJ0.
