# Supplementary material for: A novel thermostable prokaryotic fucoidan active sulfatase PsFucS1 with an unusual quaternary hexameric structure
Source: Sci Rep. 2021 Sep 30;11:19523. doi: 10.1038/s41598-021-98588-3 (PMC8484680; doi:10.1038/s41598-021-98588-3)
Supplement: Supplementary file 1 — Supplementary Information. [file 41598_2021_98588_MOESM1_ESM.pdf]

Supplementary information

A novel thermostable prokaryotic fucoidan active sulfatase PsFucS1 with an unusual quaternary hexameric structure

Maria Dalgaard Mikkelsen[1*], Hang Thi Thuy Cao[1, 2*], Thomas Roret[3], Nanna Rhein-Knudsen[1], Jesper Holck[1], Van Thi Thanh Tran[2], Thuan Thi Nguyen[1,2], Vy Tran Nguyen Ha[1], Mateusz Jakub Lezyk[1], Jan Muschiol[1], Thinh Duc Pham[2], Mirjam Czjzek[3] and Anne S. Meyer[1]**

[1]Protein Chemistry and Enzyme Technology Section, DTU Bioengineering, Department of Biotechnology and Biomedicine, Technical University of Denmark, 2800 Kgs Lyngby, Denmark.

[2]NhaTrang Institute of Technology Research and Application, Vietnam Academy of Science and Technology, 02 Hung Vuong Street, Nhatrang 650000, Socialist Republic of Vietnam.

[3]Sorbonne Université, CNRS, Integrative Biology of Marine Models, Station Biologique de Roscoff, 29680 Roscoff, France.

[*]Authors contributed equally to the work.

[**]Corresponding author, Address as above, e-mail: asme@dtu.dk

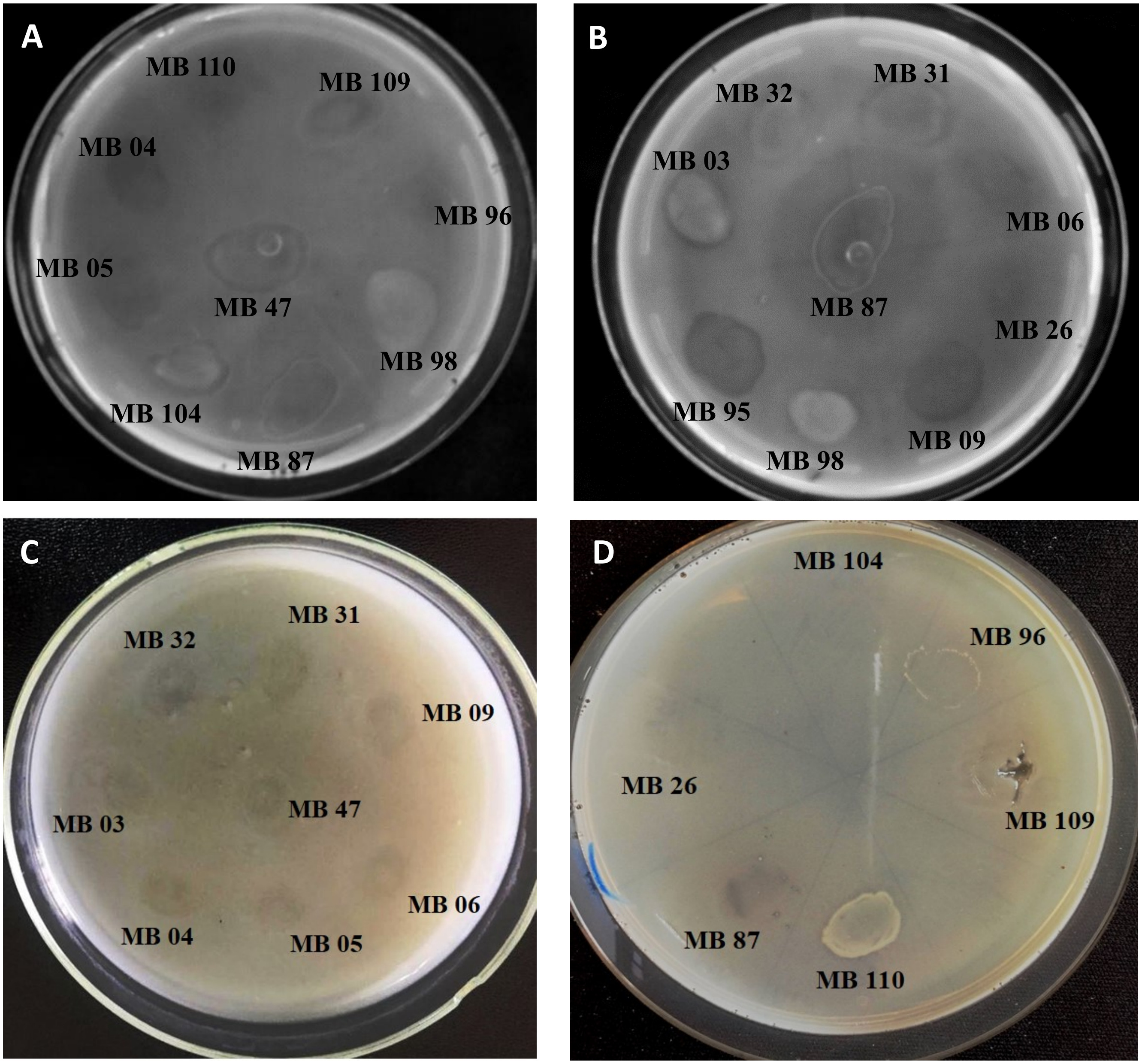

Supplementary Figure 1. Detection of bacteria producing fucoidan-modifying enzymes. Cells are grown on fucoidan-agar plates with A) and B) *S. mcclurei* or C) and D) *T. ornata* fucoidan and afterwards treated with cetavlon. The MB03, MB04, MB05, MB06, MB09, MB26, MB31, MB32, MB47, MB87, MB95, MB96, MB104, MB109, and MB110 strains showed clearance zones around the cell streaks on *S. mcclurei* fucoidan, as well as on *T. ornata* fucoidan, the latter except MB96, MB104, MB109 and MB110, indicating production of fucoidan modifying enzymes. Strain MB87 showed a very large clearance zone on S. mcclurei fucoidan, indicating high fucoidan-modifying activity of this strain. MB98: negative control strain.

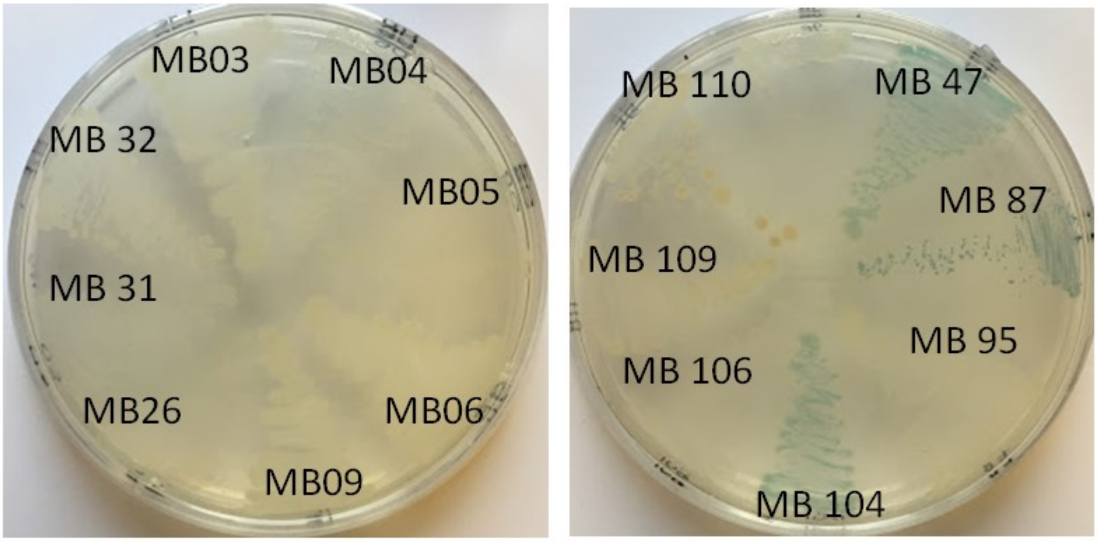

Supplementary Figure 2. Detection of bacteria producing X-SO_4 specific sulfatases. Cells were grown on agar plates containing X-SO_4, which when cleaved produces blue coloring. The MB47, MB87, and MB104 strains turned blue, indicting X-SO_4 specific sulfatase activity, while all other strains did not turn blue, indicating no X-SO_4 specific sulfatase activity.

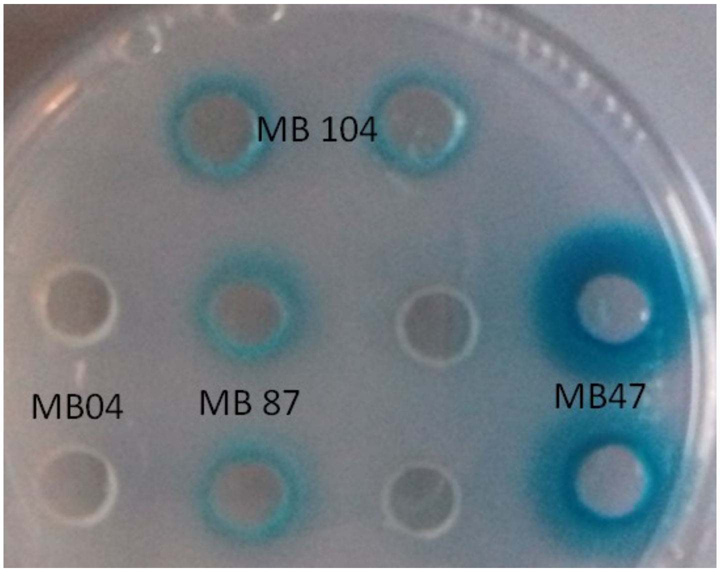

Supplementary Figure 3. Detection of bacterial cell extracts producing X-SO_4 specific sulfatases. Cells were grown on agar plates containing X-SO_4, which when cleaved produces blue coloring. Cell extracts were added to exercised holes in the plate. The MB87 and MB104 strains showed X-SO_4 specific sulfatase activity, while the MB47 strain showed comparably higher X-SO_4 specific sulfatase activity. Negative control strain: MB04.

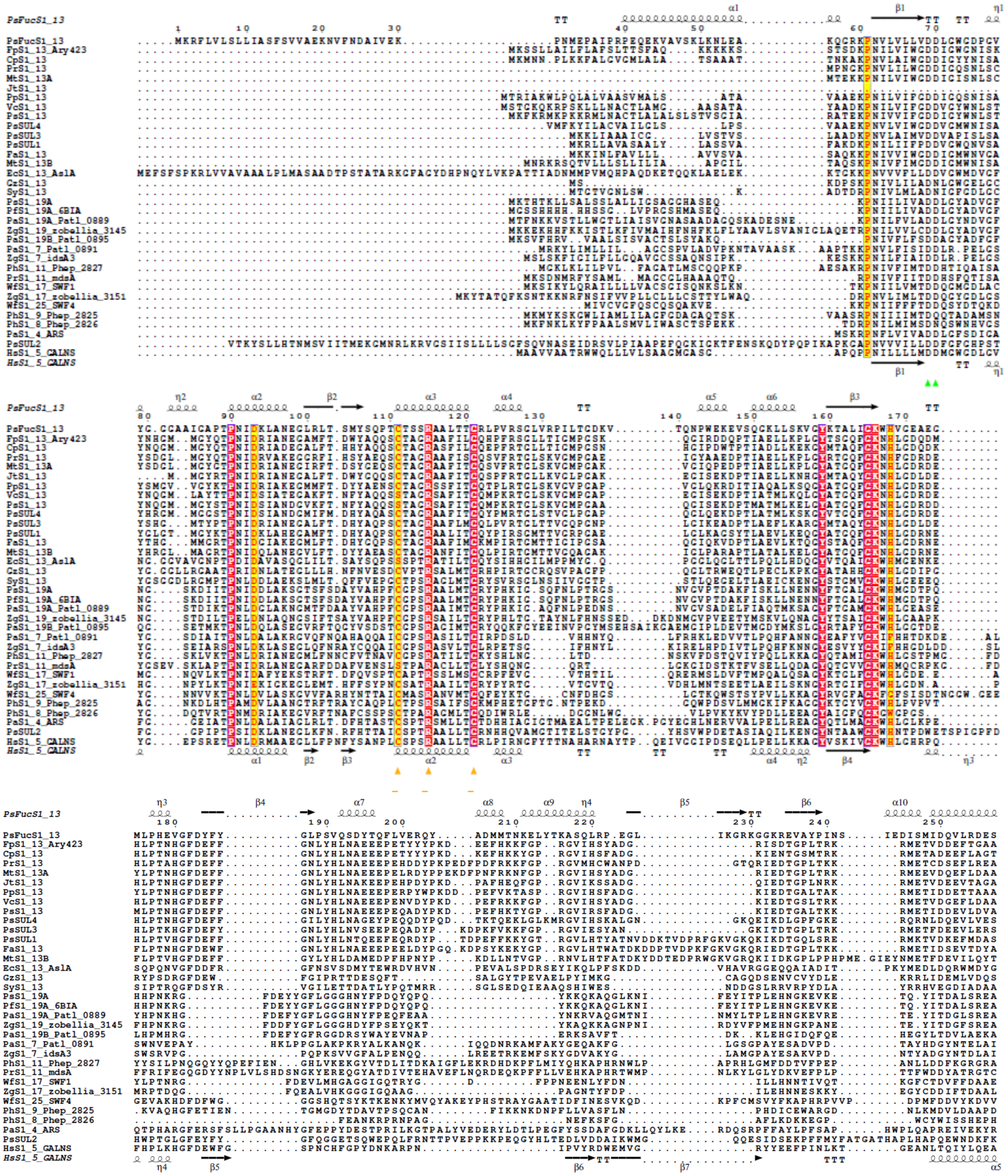

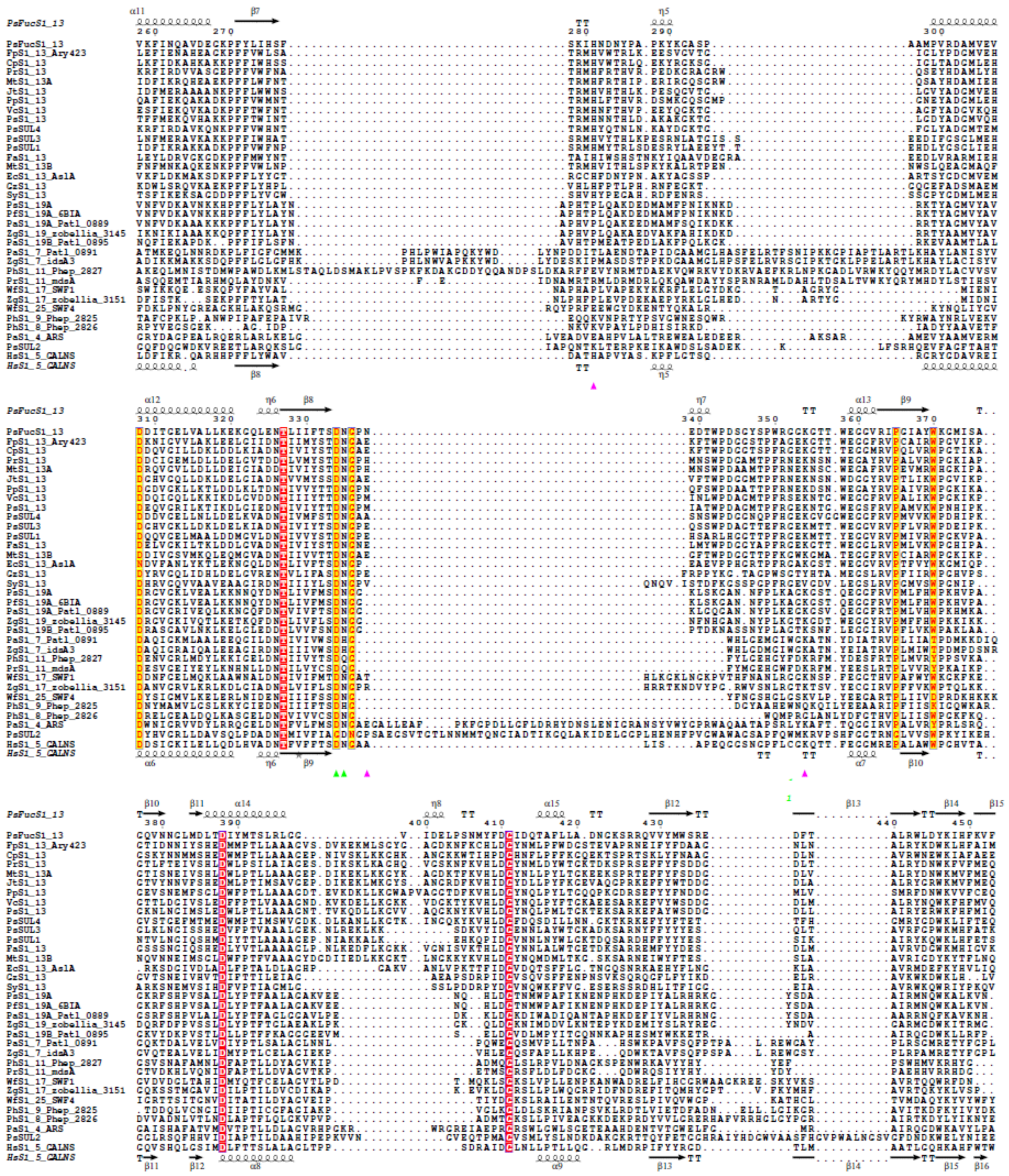

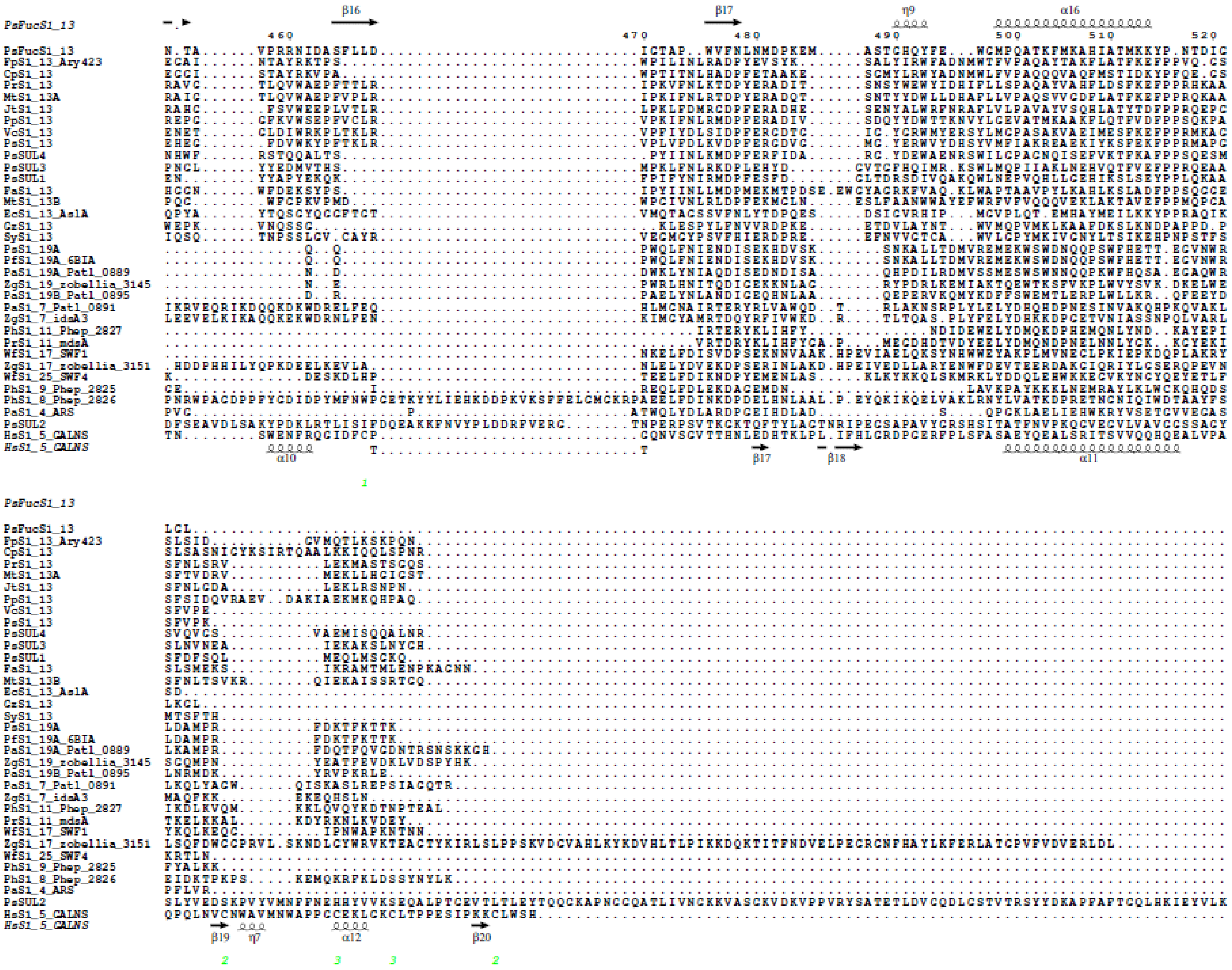

Supplementary Figure 4. Multiple alignment of selected S1 family sulfatases. List of sequences used (GenBank, UniProt or PDB accession no. in brackets): PsSUL1-4 (WP_166340718.1, WP_166338356.1, WP_054553996.1, WP_166340629.1) and PsFucS1 (WP_166338360.1) from *Pseudoalteromonas* sp. MB47 sequences, the human HsS1_5_GALNS sequence (P34059), FpS1_13_Ary432 from *Flammeovirga pacifica* (AKL72071.1), CpS1_13 from *Colwellia psycherythraea* (KGJ89632), PrS1_13 from *Prochlorococcus* sp. (WP_036912803.1), MtS1_13A from *Methanosarcina thermophila* (WP_048168313.1), JtS1_13 from *Jhaorihella thermophila* (WP_104007885.1), PpS1_13 from *Pseudomonas psychrophila* (WP_037032392.1), VcS1_13 from *Vibrio cholerae* (EEW10787.1), PsS1_13 from *Psychromonas* sp. (AGH81339.1), FaS1_13 from *Formosa agariphila* (WP_038527574.1), MtS1_13B from *M. thermophila* (WP_067152985.1), EcS1_13_AslA from *Escherichia coli* (WP_000395863.1), GzS1_13 from *Gibberella zeae* (I1S2W7), SyS1_13 from *Synechococcus* sp. (WP_067326625.1), PsS1_19A from *Pseudoalteromonas* sp. PS47 (AWF93649.1),

PfS1_19A_6BIA from *Pseudoalteromonas fuliginea* (6BIA), PaS1_19A_Patl_0889 from *Pseudoalteromonas atlantica* (Q15XH3), ZgS1_19_zobellia_3145 from *Zobellia galactanivorans* (G0L000), PaS1_19B_Patl_0895 from *P. atlantica* (Q15XG7), PaS1_7_Patl_0891 from *P. atlantica* (Q15XH1), ZgS1_7_idsA3 from *Z. galactanivorans* (G0L7B4), PhS1_11_Phep_2827 from *Pedobacter heparinus* (C6Y1N4), PrS1_11_mdsA from *Prevotella* sp. (Q9L5W0), WfS1_17_SWF1 from *Wenyingzhuangia fucanilytica* (WP_068825883.1), ZgS1_17_zobellia_3151 from *Z. galactanivorans* (G0L7B6), WfS1_25_SWF4 from *W. fucanilytica* (WP_068828765.1), PhS1_9_Phep_2825 from *P. heparinus* (C6Y1N2), PhS1_8_Phep_2826 from *P. heparinus* (C6Y1N3), PaS1_4_ARS from *Pseudomonas aeruginosa* (P51691). β-sheets (arrows) and α-helixes (helical) are indicated for PsFucS1 above the alignment and for GALNS below the alignment. Triangular marks below the alignment: Yellow: Conserved active site CXXXRXXXXXG. Green: Amino acids involved in binding of the active site metal ion. Pink: Residues involved in substrate coordination in PsFucS1.

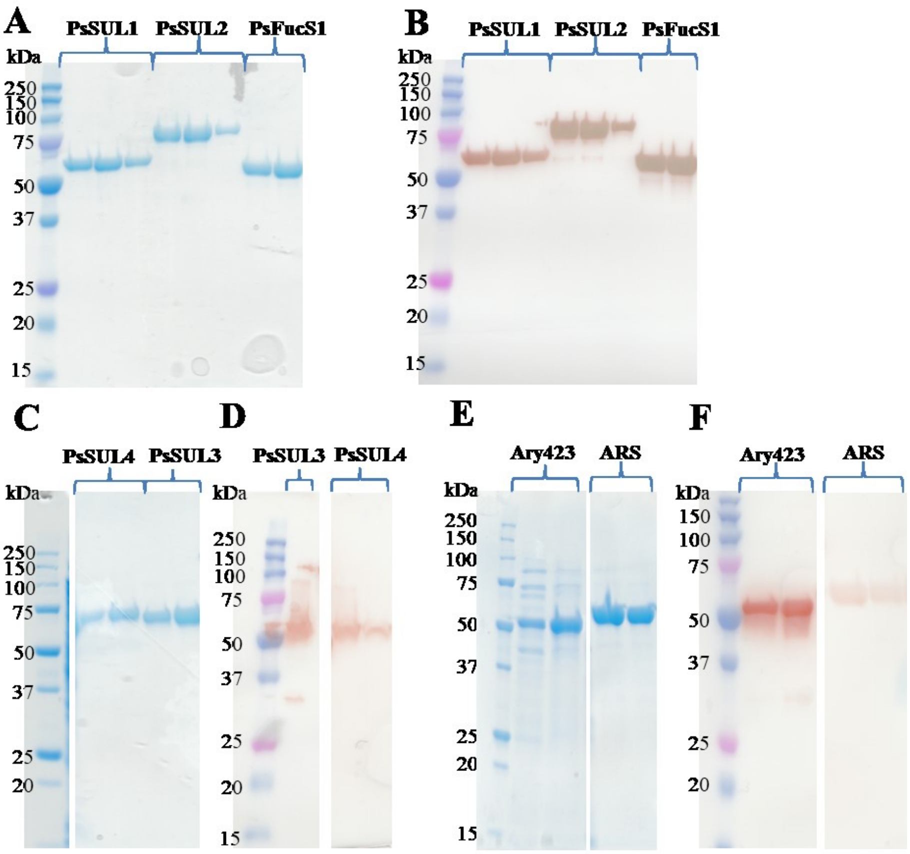

Supplementary Figure 5. Purified recombinantly expressed sulfatases. A), C) and E) SDS-PAGE and B), D) and F) western blots of PsSUL1: 57 kDa, PsSUL2: 90 kDa, PsSUL3: 55 kDa, PsSUL4: 56 kDa, PsFucS1: 57 kDa, Ary423: 56 kDa, and ARS: 61 kDa, using poly-histidine antibodies. For SDS-PAGE the protein plus molecular weight marker was used, while for western blot analysis the prestained marker was used. All proteins had the expected sizes.

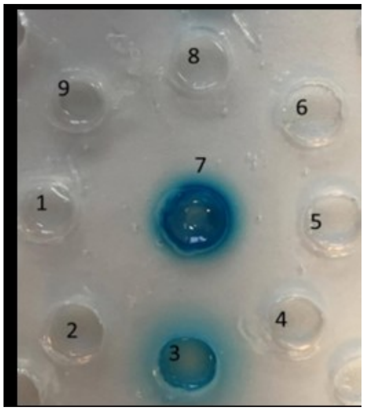

Supplementary Figure 6. Sulfatase activity of *E. coli* crude extracts. *E. coli* crude extracts from containing constructs containing 1) PsSUL1, 2) PsSUL2, 3) PsFucS1, 4) PsSUL3, 5) Ps3148, 6) PsSUL4, 7) ARS, 8) Ary432, and 9) AER35705 was applied to excised holes in X-SO_4 containing agar plates. ARS and PsFucS1 showed activity by formation of blue color due to release of sulfate.

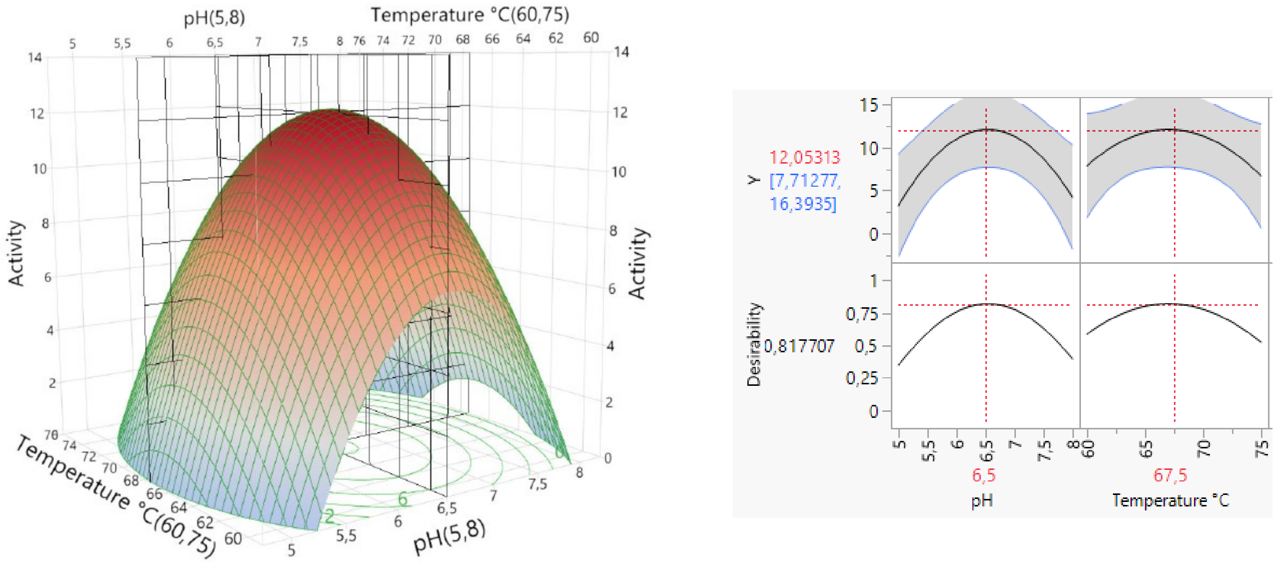

Reaction	pH	Temperature °C	Activity µM pNCS converted/min· mg	s.d.
1	5	60	0.0645	0.0129
2	8	60	1.2358	0.0999
3	5	75	0.0559	0.0074
4	8	75	0.2751	0.0268
5	5	67.5	0.0236	0.0074
6	8	67.5	1.7128	0.0998
7	6.5	60	5.6671	0.2872
8	6.5	75	3.1827	0.0492
9 cp	6.5	67.5	14.338	} 0.4708
10 cp	6.5	67.5	13.436	
11 cp	6.5	67.5	14.119	

Supplementary Figure 7. Surface response plot of PsFucS1 sulfatase activity as a function of temperature and pH; Data details for each reaction in the composite design. The buffer controlled incubation mixture contained 10 mM Ca^{2+}, 125 mM NaCl, and 2.5 mM pNCS. The color grading indicates predicted activity level based on activity data in a CCF experimental design: blue (low sulfatase activity) to red (high sulfatase activity). Based on these data the optimal reaction conditions of the PsFucS1 sulfatase was pH 6.5 and 68 °C (67.5 °C) giving a predicted activity of ~12 U.

U = µM pNCS converted/min · mg enzyme. All reactions were run in triplicate, in a random reaction order; cp: repeated center point.

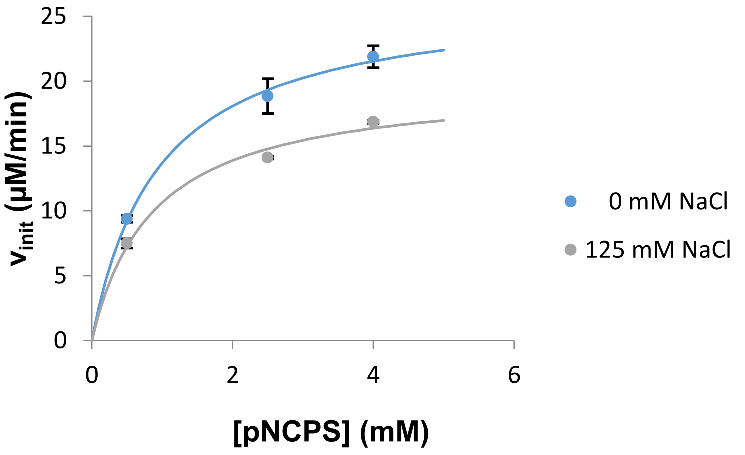

NaCl (mM)	Apparent K_M (mM)	Apparent V_{max} (μM_{pNCP}/min·mg)
0	0.95 ± 0.15	26.6 ± 1.2
125	0.88 ± 0.25	19.9 ± 1.6

Supplementary Figure 8. Kinetics of the recombinant PsFucS1 sulfatase. The kinetic parameters of PsFucS1 was determined on the pNCS substrate in presence (125 mM) or absence of NaCl. The apparent K_M and V_{max} were slightly reduced in the presence of 125 mM NaCl.

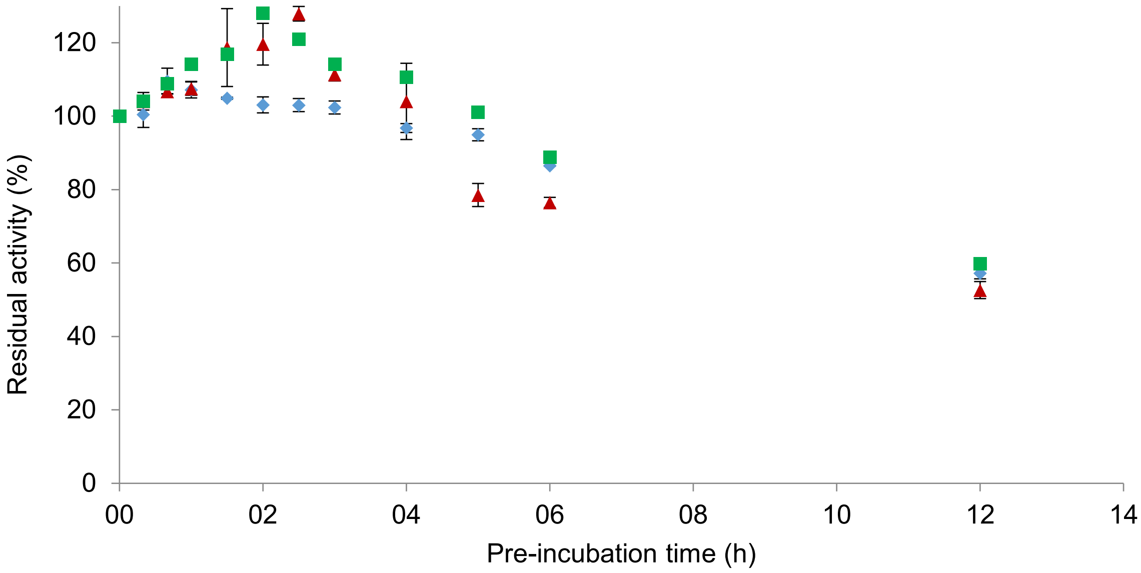

Supplementary Figure 9. Thermostability of the recombinant PsFucS1 sulfatase. The residual activity of PsFucS1 was determined by assaying the samples taken at regular time intervals on pNCS at 68°C during pre-incubation of PsFucS1 for 12 hours. As evident, PsFucS1 retained almost 60% of its maximum activity after 12 hours of incubation at 68°C even when incubated in the presence of 62.5 mM or 125 mM NaCl. Light blue diamonds: 0 mM NaCl in the pre-incubation mix; Green squares: With 62.5 mM NaCl in the pre-incubation mixture; Red triangles: With 125 mM NaCl in the pre-incubation mixture. Data shown are averages of replicate incubations and shown ± s.d.

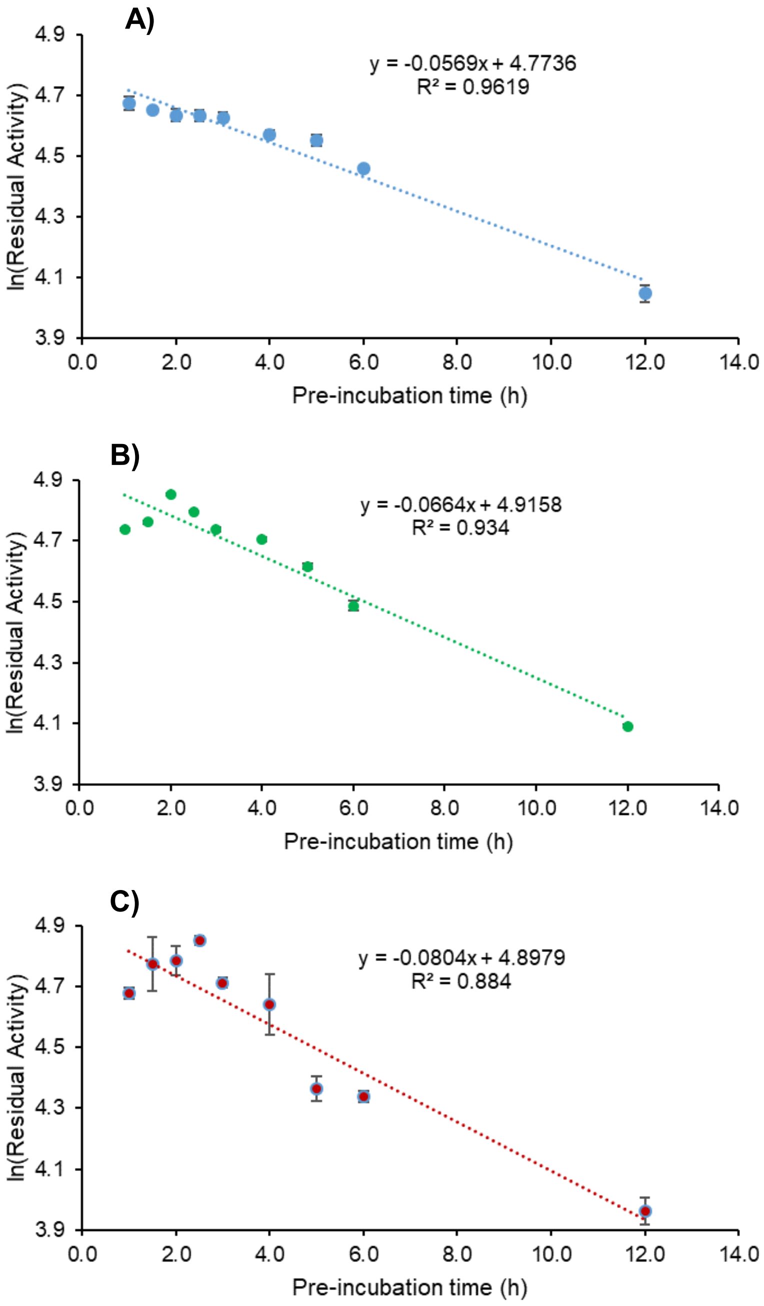

Supplementary Figure 10. Thermal stability of the recombinant PsFucS1. Semi logarithmic linear plots obtained for residual activity (%) vs. pre-incubation time (data shown in Supplementary Figure 9) after: A) Pre-incubation without NaCl, B) Pre-incubation in presence of 62.5 mM NaCl, C) Pre-incubation in presence of 125 mM NaCl. The residual activity of PsFucS1 was monitored by assaying the samples at 68°C on pNCS in the standard pNCS colorimetric assay described in methods. The first order rate constant of the thermal denaturation (k_D) was obtained from the slope of the linear regression lines. Data shown are averages of replicate incubations ± s.d.

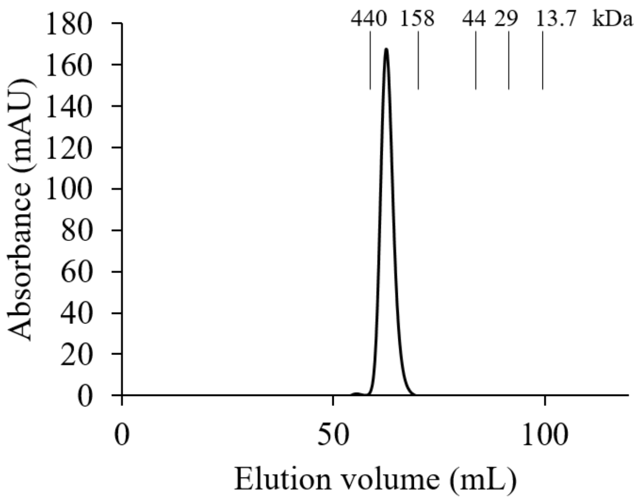

Supplementary Figure 11. Size-exclusion chromatography of PsFucS1. The representative chromatogram was obtained for PsFucS1 by recording absorbance at 280 nm after analytical size-exclusion chromatography performed using a Superdex 200 16/600 column. The approximate size of the native PsFucS1 in solution was estimated to approximately 291 kDa, corresponding to the approximate size of a hexamer (calculated to be 345.8 kDa).

Supplementary Table 1. 16S DNA analysis of sea cucumber gut bacterium.

Strain	Query coverage	Homolog id	Scientific name (homolog)	Accession
MB47	100.00%	99.87%	*Pseudoalteromonas shioyasakiensis*	NR_125458.1

Supplementary Table 2. Draft genome assembly of strain MB47.

Strain	Genera/species	Completeness (%)	Size (bp)	No. contigs	GC (%)	CDS	tRNAs
MB47	*Pseudoalteromonas* sp.	100.0	4,545,585	31	41.1	4017	94

Supplementary Table 3. Overview of putative carbohydrate modifying enzymes and carbohydrate binding modules (including CAZy family affiliation) identified from the genome of *Pseudoalteromonas* sp. MB47 with dbCAN models. AA: Auxiliary Activity; CBM: Carbohydrate-binding module; CE: Carbohydrate Esterase; PL: Polysaccharide Lyase. GH: Glycoside Hydrolase; GT: Glycosyl Transferase.

CAZy family		Activities
AA2	2	Manganese peroxidase (EC 1.11.1.13); versatile peroxidase (EC 1.11.1.16); lignin peroxidase (EC 1.11.1.14); peroxidase (EC 1.11.1.-); cytochrome-c peroxidase (EC 1.11.1.5); ascorbate peroxidase (EC 1.11.1.11)
AA3	4	Cellobiose dehydrogenase (EC 1.1.99.18); glucose 1-oxidase (EC 1.1.3.4); aryl alcohol oxidase (EC 1.1.3.7); alcohol oxidase (EC 1.1.3.13); pyranose oxidase (EC 1.1.3.10)
AA6	3	1,4-benzoquinone reductase (EC. 1.6.5.6)
AA10	1	AA10 (formerly CBM33) proteins are copper-dependent lytic polysaccharide monooxygenases (LPMOs); some proteins have been shown to act on chitin, others on cellulose; lytic cellulose monooxygenase (C1-hydroxylating) (EC 1.14.99.54); lytic cellulose monooxygenase (C4-dehydrogenating)(EC 1.14.99.56); lytic chitin monooxygenase (EC 1.14.99.53); lytic xylan monooxygenase / xylan oxidase (glycosidic bond-cleaving) (EC 1.14.99.-)
AA12	1	The pyrroloquinoline quinone-dependent oxidoreductase activity was demonstrated for the CC1G_09525 protein of *Coprinopsis cinerea*.
CBM4	1	Modules of approx. 150 residues found in bacterial enzymes. Binding of these modules has been demonstrated with xylan, β-1,3-glucan, β-1,3-1,4-glucan, β-1,6-glucan and amorphous cellulose but not with crystalline cellulose.
CBM5	4	Modules of approx. 60 residues found in bacterial enzymes. Chitin-binding described in several cases. Distantly related to the CBM12 family.
CBM9	3	Modules of approx. 170 residues found so far only in xylanases. The cellulose-binding function has been demonstrated in one case.
CBM13	3	Modules of approx. 150 residues which always appear as a threefold internal repeat. The only apparent exception to this, xylanase II of Actinomadura sp. FC7 (GenBank U08894), is in fact not completely sequenced. These modules were first identified in several plant lectins such as ricin or agglutinin of Ricinus communis which bind galactose residues. The three-dimensional structure of a plant lectin has been determined and displays a pseudo-threefold symmetry in accord with the observed sequence threefold repeat. These modules have since been found in a number of other proteins of various functions including glycoside hydrolases and glycosyltransferases. While in the plant lectins this module binds mannose, binding to xylan has been demonstrated in the Streptomyces lividans xylanase A and arabinofuranosidase B. Binding to GalNAc has been shown for the corresponding module of GalNAc transferase 4. For the other proteins, the binding specificity of these modules has not been established. The pseudo three-fold symmetry of the CBM13 module has now been confirmed in the 3-D structure of the intact, two-domain, xylanase of *Streptomyces olivaceoviridis*.
CBM16	1	Carbohydrate-binding module 16. Binding to cellulose and glucomannan
CBM32	2	Binding to galactose and lactose has been demonstrated for the module of Micromonospora viridifaciens sialidase (PMID: 16239725). Binding to polygalacturonic acid has been shown for a Yersinia member (PMID: 17292916). Binding to LacNAc (beta-D-galactosyl-1,4-beta-D-N-acetylglucosamine) has been shown for an N-acetylglucosaminidase from Clostridium perfingens (PMID: 16990278).
CBM35	1	Modules of approx. 130 residues. A module that is conserved in three Cellvibrio xylan-degrading enzymes binds to xylan and the interaction is calcium dependent, while a module from a Cellvibrio mannanase binds to decorated soluble mannans and mannooligosaccharides. A module in a

CBM40	1	Modules of approx. 200 residues, found at the N-terminus of GH33 sialidases. Can also be found inserted in the beta-propeller of GH33 sialidases. The sialic acid binding function has been demonstrated for the N-terminal CBM40 of Vibrio cholerae sialidase
CBM41	1	Modules of approx. 100 residues found in primarily in bacterial pullulanases. The N-terminal module from Thermotoga maritima Pul13 has been shown to bind to the alpha-glucans amylose, amylopectin, pullulan, and oligosaccharide fragments derived from these polysaccharides
CBM44	1	The C-terminal CBM44 module of the Clostridium thermocellum enzyme has been demonstrated to bind equally well cellulose and xyloglucan
CBM48	4	Modules of approx. 100 residues with glycogen-binding function, appended to GH13 modules. Also found in the beta subunit (glycogen-binding) of AMP-activated protein kinases (AMPK)
CBM50	8	Modules of approx. 50 residues found attached to various enzymes from families GH18, GH19, GH23, GH24, GH25 and GH73, i.e. enzymes cleaving either chitin or peptidoglycan. Binding to chitopentaose demonstrated in the case of Pteris ryukyuensis chitinase A. CBM50 modules are also found in a multitude of other enzymes targeting the petidoglycan such as peptidases and amidases. These enzymes are not reported in the list below.
CBM66	1	The CBM66 module, derived from the Bacillus subtilis exo-acting beta-fructosidase SacC, targets the terminal fructoside residue of fructans.
CBM69	1	Starch-binding function demonstrated in one case; distantly related to families CBM20 and CBM48
CBM70	1	The hyaluronan-specific binding function of the N-terminal CBM70 module of Streptococcus pneumoniae hyaluronate lyase has been demonstrated.
CBM73	1	Modules of approx 65 residues found on various enzymes active of chitin. Chitin-binding function demonstrated for the Cellvibrio japonicus CjLPMO10A protein.
CE1	18	Acetyl xylan esterase (EC 3.1.1.72); cinnamoyl esterase (EC 3.1.1.-); feruloyl esterase (EC 3.1.1.73); carboxylesterase (EC 3.1.1.1); S-formylglutathione hydrolase (EC 3.1.2.12); diacylglycerol O-acyltransferase (EC 2.3.1.20); trehalose 6-O-mycolyltransferase (EC 2.3.1.122)
CE3	1	Acetyl xylan esterase (EC 3.1.1.72).
CE4	2	Acetyl xylan esterase (EC 3.1.1.72); chitin deacetylase (EC 3.5.1.41); chitooligosaccharide deacetylase (EC 3.5.1.-); peptidoglycan GlcNAc deacetylase (EC 3.5.1.-); peptidoglycan N-acetylmuramic acid deacetylase (EC 3.5.1.-).
CE6	1	Acetyl xylan esterase (EC 3.1.1.72).
CE9	3	N-acetylglucosamine 6-phosphate deacetylase (EC 3.5.1.25); N-acetylglucosamine 6-phosphate deacetylase (EC 3.5.1.80)
CE10	14	Arylesterase (EC 3.1.1.-); carboxyl esterase (EC 3.1.1.3); acetylcholinesterase (EC 3.1.1.7); cholinesterase (EC 3.1.1.8); sterol esterase (EC 3.1.1.13); brefeldin A esterase (EC 3.1.1.-).
CE11	1	UDP-3-0-acyl N-acetylglucosamine deacetylase (EC 3.5.1.108).
GH1	1	Beta-glucosidase (EC 3.2.1.21); beta-galactosidase (EC 3.2.1.23); beta-mannosidase (EC 3.2.1.25); beta-glucuronidase (EC 3.2.1.31); beta-xylosidase (EC 3.2.1.37); beta-D-fucosidase (EC 3.2.1.38); phlorizin hydrolase (EC 3.2.1.62); exo-beta-1,4-glucanase (EC 3.2.1.74); 6-phospho-beta-galactosidase (EC 3.2.1.85); 6-phospho-beta-glucosidase (EC 3.2.1.86); strictosidine beta-glucosidase (EC 3.2.1.105); lactase (EC 3.2.1.108); amygdalin beta-glucosidase (EC 3.2.1.117); prunasin beta-glucosidase (EC 3.2.1.118); vicianin hydrolase (EC 3.2.1.119); raucaffricine beta-glucosidase (EC 3.2.1.125); thioglucosidase (EC 3.2.1.147); beta-primeverosidase (EC 3.2.1.149); isoflavonoid 7-O-beta-apiosyl-beta-glucosidase (EC 3.2.1.161); ABA-specific beta-glucosidase (EC 3.2.1.175); DIMBOA beta-glucosidase (EC 3.2.1.182); beta-glycosidase (EC 3.2.1.-); hydroxyisourate hydrolase (EC 3.-.-.-)
GH2	1	Beta-galactosidase (EC 3.2.1.23) ; beta-mannosidase (EC 3.2.1.25); beta-glucuronidase (EC 3.2.1.31); alpha-L-arabinofuranosidase (EC 3.2.1.55); mannosylglycoprotein endo-beta-mannosidase (EC 3.2.1.152); exo-beta-glucosaminidase (EC 3.2.1.165); alpha-L-arabinopyranosidase (EC 3.2.1.-); beta-galacturonidase (EC 3.2.1.-); beta-xylosidase (EC 3.2.1.37); beta-D-galactofuranosidase (EC 3.2.1.146);
GH3	2	Beta-glucosidase (EC 3.2.1.21); xylan 1,4-beta-xylosidase (EC 3.2.1.37); beta-glucosylceramidase (EC 3.2.1.45); beta-N-acetylhexosaminidase (EC 3.2.1.52); alpha-L-arabinofuranosidase (EC 3.2.1.55); glucan 1,3-beta-glucosidase (EC 3.2.1.58); glucan 1,4-beta-glucosidase (EC 3.2.1.74); isoprimeverose-producing oligoxyloglucan hydrolase (EC 3.2.1.120); coniferin beta-glucosidase (EC

		3.2.1.126); exo-1,3-1,4-glucanase (EC 3.2.1.-); beta-N-acetylglucosaminide phosphorylases (EC 2.4.1.-)
GH4	1	Maltose-6-phosphate glucosidase (EC 3.2.1.122); alpha-glucosidase (EC 3.2.1.20); alpha-galactosidase (EC 3.2.1.22); 6-phospho-beta-glucosidase (EC 3.2.1.86); alpha-glucuronidase (EC 3.2.1.139); alpha-galacturonase (EC 3.2.1.67); palatinase (EC 3.2.1.-)
GH13	8	Alpha-amylase (EC 3.2.1.1); pullulanase (EC 3.2.1.41); cyclomaltodextrin glucanotransferase (EC 2.4.1.19); cyclomaltodextrinase (EC 3.2.1.54); trehalose-6-phosphate hydrolase (EC 3.2.1.93); oligo-alpha-glucosidase (EC 3.2.1.10); maltogenic amylase (EC 3.2.1.133); neopullulanase (EC 3.2.1.135); alpha-glucosidase (EC 3.2.1.20); maltotetraose-forming alpha-amylase (EC 3.2.1.60); isoamylase (EC 3.2.1.68); glucodextranase (EC 3.2.1.70); maltohexaose-forming alpha-amylase (EC 3.2.1.98); maltotriose-forming alpha-amylase (EC 3.2.1.116); branching enzyme (EC 2.4.1.18); trehalose synthase (EC 5.4.99.16); 4-alpha-glucanotransferase (EC 2.4.1.25); maltopentaose-forming alpha-amylase (EC 3.2.1.-) ; amylosucrase (EC 2.4.1.4) ; sucrose phosphorylase (EC 2.4.1.7); malto-oligosyltrehalose trehalohydrolase (EC 3.2.1.141); isomaltulose synthase (EC 5.4.99.11); malto-oligosyltrehalose synthase (EC 5.4.99.15); amylo-alpha-1,6-glucosidase (EC 3.2.1.33); alpha-1,4-glucan: phosphate alpha-maltosyltransferase (EC 2.4.99.16); 6'-P-sucrose phosphorylase (EC 2.4.1.-); amino acid transporter
GH15	1	Glucoamylase (EC 3.2.1.3); glucodextranase (EC 3.2.1.70); alpha,alpha-trehalase (EC 3.2.1.28); dextran dextrinase (EC 2.4.1.2)
GH16	2	Xyloglucan:xyloglucosyltransferase (EC 2.4.1.207); keratan-sulfate endo-1,4-beta-galactosidase (EC 3.2.1.103); endo-1,3-beta-glucanase / laminarinase (EC 3.2.1.39); endo-1,3(4)-beta-glucanase (EC 3.2.1.6); licheninase (EC 3.2.1.73); beta-agarase (EC 3.2.1.81); kappa;-carrageenase (EC 3.2.1.83); xyloglucanase (EC 3.2.1.151); endo-beta-1,3-galactanase (EC 3.2.1.181); [retaining] beta-porphyranase (EC 3.2.1.178); hyaluronidase (EC 3.2.1.35); endo-beta-1,4-galactosidase (EC 3.2.1.-); chitin beta-1,6-glucanosyltransferase (EC 2.4.1.-); beta-transglycosidase (EC 2.4.1.-); beta-glycosidase (EC 3.2.1.-); endo-beta-1,3-galactanase (EC 3.2.1.181)
GH18	2	Chitinase (EC 3.2.1.14); lysozyme (EC 3.2.1.17); endo-beta-N-acetylglucosaminidase (EC 3.2.1.96); peptidoglycan hydrolase with endo-beta-N-acetylglucosaminidase specificity (EC 3.2.1.-); Nod factor hydrolase (EC 3.2.1.-); xylanase inhibitor; concanavalin B; narbonin
GH20	2	Beta-hexosaminidase (EC 3.2.1.52); lacto-N-biosidase (EC 3.2.1.140); beta-1,6-N-acetylglucosaminidase (EC 3.2.1.-); beta-6-SO3-N-acetylglucosaminidase (EC 3.2.1.-)
GH23	6	Lysozyme type G (EC 3.2.1.17); peptidoglycan lyase (EC 4.2.2.n1) also known in the literature as peptidoglycan lytic transglycosylase; chitinase (EC 3.2.1.14)
GH24	1	Lysozyme (EC 3.2.1.17)
GH37	1	Alpha,alpha-trehalase (EC 3.2.1.28).
GH43	7	Beta-xylosidase (EC 3.2.1.37); alpha-L-arabinofuranosidase (EC 3.2.1.55); xylanase (EC 3.2.1.8); alpha-1,2-L-arabinofuranosidase (EC 3.2.1.-); exo-alpha-1,5-L-arabinofuranosidase (EC 3.2.1.-); [inverting] exo-alpha-1,5-L-arabinanase (EC 3.2.1.-); beta-1,3-xylosidase (EC 3.2.1.-); [inverting] exo-alpha-1,5-L-arabinanase (EC 3.2.1.-); [inverting] endo-alpha-1,5-L-arabinanase (EC 3.2.1.99); exo-beta-1,3-galactanase (EC 3.2.1.145); beta-D-galactofuranosidase (EC 3.2.1.146)
GH73	2	Lysozyme (EC 3.2.1.17); mannosyl-glycoprotein endo-beta-N-acetylglucosaminidase (EC 3.2.1.96); peptidoglycan hydrolase with endo-beta-N-acetylglucosaminidase specificity (EC 3.2.1.-)
GH76	1	Alpha-1,6-mannanase (EC 3.2.1.101); alpha-glucosidase (EC 3.2.1.20)
GH77	1	Amylomaltase or 4-alpha-glucanotransferase (EC 2.4.1.25)
GH97	2	Glucoamylase (EC 3.2.1.3); alpha-glucosidase (EC 3.2.1.20); alpha-galactosidase (EC 3.2.1.22)
GH103	1	Peptidoglycan lytic transglycosylase (EC 3.2.1.-)
GH109	4	Alpha-N-acetylgalactosaminidase (EC 3.2.1.49)
GH127	1	Beta-L-arabinofuranosidase (EC 3.2.1.185); 3-C-carboxy-5-deoxy-L-xylose (aceric acid) hydrolase (EC 3.2.1.-)
GH135	1	Alpha-1,4-galactosaminogalactan hydrolase (EC 3.2.1.-)
GH145	1	L-Rhalpha-alpha-1,4-GlcA alpha-L-rhamnohydrolase (EC 3.2.1.-)
GT2	7	Cellulose synthase (EC 2.4.1.12); chitin synthase (EC 2.4.1.16); dolichyl-phosphate beta-D-mannosyltransferase (EC 2.4.1.83); dolichyl-phosphate beta-glucosyltransferase (EC 2.4.1.117); N-acetylglucosaminyltransferase (EC 2.4.1.-); N-acetylgalactosaminyltransferase (EC 2.4.1.-);

		hyaluronan synthase (EC 2.4.1.212); chitin oligosaccharide synthase (EC 2.4.1.-); beta-1,3-glucan synthase (EC 2.4.1.34); beta-1,4-mannan synthase (EC 2.4.1.-); beta-mannosylphosphodecaprenol-mannooligosaccharide alpha-1,6-mannosyltransferase (EC 2.4.1.199); UDP-Galf: rhamnopyranosyl-N-acetylglucosaminyl-PP-decaprenol beta-1,4/1,5-galactofuranosyltransferase (EC 2.4.1.287); UDP-Galf: galactofuranosyl-galactofuranosyl-rhamnosyl-N-acetylglucosaminyl-PP-decaprenol beta-1,5/1,6-galactofuranosyltransferase (EC 2.4.1.288); dTDP-L-Rha: N-acetylglucosaminyl-PP-decaprenol alpha-1,3-L-rhamnosyltransferase (EC 2.4.1.289)
GT4	9	Sucrose synthase (EC 2.4.1.13); sucrose-phosphate synthase (EC 2.4.1.14); alpha-glucosyltransferase (EC 2.4.1.52); lipopolysaccharide N-acetylglucosaminyltransferase (EC 2.4.1.56); phosphatidylinositol alpha-mannosyltransferase (EC 2.4.1.57); GDP-Man: Man1GlcNAc2-PP-dolichol alpha-1,3-mannosyltransferase (EC 2.4.1.132); GDP-Man: Man3GlcNAc2-PP-dolichol/Man4GlcNAc2-PP-dolichol alpha-1,2-mannosyltransferase (EC 2.4.1.131); digalactosyldiacylglycerol synthase (EC 2.4.1.141); 1,2-diacylglycerol 3-glucosyltransferase (EC 2.4.1.157); diglucosyl diacylglycerol synthase (EC 2.4.1.208); trehalose phosphorylase (EC 2.4.1.231); NDP-Glc: alpha-glucose alpha-glucosyltransferase / alpha,alpha-trehalose synthase (EC 2.4.1.245); GDP-Man: Man2GlcNAc2-PP-dolichol alpha-1,6-mannosyltransferase (EC 2.4.1.257); UDP-GlcNAc: 2-deoxystreptamine alpha-N-acetylglucosaminyltransferase (EC 2.4.1.283); UDP-GlcNAc: ribostamycin alpha-N-acetylglucosaminyltransferase (EC 2.4.1.285); UDP-Gal alpha-galactosyltransferase (EC 2.4.1.-); UDP-Xyl alpha-xylosyltransferase (EC 2.4.2.-); UDP-GlcA alpha-glucuronyltransferase (EC 2.4.1.-); UDP-Glc alpha-glucosyltransferase (EC 2.4.1.-); UDP-GalNAc: GalNAc-PP-Und alpha-1,3-N-acetylgalactosaminyltransferase (EC 2.4.1.306); UDP-GalNAc: N,N'-diacetylbacillosaminyl-PP-Und alpha-1,3-N-acetylgalactosaminyltransferase (EC 2.4.1.290); ADP-dependent alpha-maltose-1-phosphate synthase (2.4.1.342)
GT5	1	UDP-Glc: glycogen glucosyltransferase (EC 2.4.1.11); ADP-Glc: starch glucosyltransferase (EC 2.4.1.21); NDP-Glc: starch glucosyltransferase (EC 2.4.1.242); UDP-Glc: alpha-1,3-glucan synthase (EC 2.4.1.183) UDP-Glc: alpha-1,4-glucan synthase (EC 2.4.1.-)
GT9	2	Lipopolysaccharide N-acetylglucosaminyltransferase (EC 2.4.1.56); heptosyltransferase (EC 2.4.-.-).
GT19	1	Lipid-A-disaccharide synthase (EC 2.4.1.182).
GT25	1	Lipopolysaccharide beta-1,4-galactosyltransferase (EC 2.4.1.-); beta-1,3-glucosyltransferase (EC 2.4.1.-); beta-1,2-glucosyltransferase (EC 2.4.1.-); beta-1,2-galactosyltransferase (EC 2.4.1.-); LPS beta-1,4-galactosyltransferase (EC 2.4.1.-); occidiofungin beta-xylosyltransferase (EC 2.4.2.-); UDP-Gal:procollagen beta-galactosyltransferase (EC 2.4.1.50)
GT26	2	UDP-ManNAcA: beta-N-acetyl mannosaminuronyltransferase (EC 2.4.1.-); UDP-ManNAc: beta-N-acetyl-mannosaminyltransferase (EC 2.4.1.-); UDP-Glc: beta-1,4-glucosyltransferase (EC 2.4.1.-); beta-1,4-galactosyltransferase (EC 2.4.1.-)
GT28	2	1,2-diacylglycerol 3-beta-galactosyltransferase (EC 2.4.1.46); 1,2-diacylglycerol 3-beta-glucosyltransferase (EC 2.4.1.157); UDP-GlcNAc: Und-PP-MurAc-pentapeptide beta-N-acetylglucosaminyltransferase (EC 2.4.1.227); digalactosyldiacylglycerol synthase (EC 2.4.1.241)
GT30	1	CMP-beta-KDO: alpha-3-deoxy-D-manno-octulosonic-acid (KDO) transferase (EC 2.4.99.-).
GT35	1	Glycogen or starch phosphorylase (EC 2.4.1.1).
GT51	2	Murein polymerase (EC 2.4.1.129).
GT70	1	UDP-GlcA: beta-glucuronosyltransferase (EC 2.4.1.17)
GT81	1	NDP-Glc: glucosyl-3-phosphoglycerate synthase (EC 2.4.1.-); NDP-Man: mannosyl-3-phosphoglycerate synthase (EC 2.4.1.-);
PL1	1	Pectate lyase (EC 4.2.2.2); exo-pectate lyase (EC 4.2.2.9); pectin lyase (EC 4.2.2.10).
PL6	2	Alginate lyase (EC 4.2.2.3); chondroitinase B (EC 4.2.2.19); MG-specific alginate lyase (EC 4.2.2.-); poly(alpha-L-guluronate) lyase / G-specific alginate lyase (EC 4.2.2.11);
PL7	1	Poly(beta-mannuronate) lyase / M-specific alginate lyase (EC 4.2.2.3); alpha-L-guluronate lyase / G-specific alginate lyase (EC 4.2.2.11); poly-(MG)-lyase / MG-specific alginate lyase (EC 4.2.2.-); endo-beta-1,4-glucuronan lyase (EC 4.2.2.14)
PL17	1	Alginate lyase (EC 4.2.2.3); oligoalginate lyase (EC 4.2.2.26)
PL22	8	Oligogalacturonate lyase / oligogalacturonide lyase (EC 4.2.2.6)

Supplementary Table 4. Predicted protein coding features with sulfatase signatures detected by InterProScan and sulfatase family groupings, S1_4 and S1_13, based on the SulfAtlas database (v1.2; http://abims.sb-roscoff.fr/sulfatlas).

Name and Subfamily group	Protein accession	Locus tag	Protein Sequence Length	Analysis	Signature Accession	Signature Description	Start location	Stop location	Score	InterPro annotation accession	InterPro annotations	GO annotations
PsFucS1 S1_13	NHH87780.1	IMPJCBKJ_00307	524	Pfam	PF00884	Sulfatase	61	399	1.2E-060	IPR000917	Sulfatase, N-terminal	GO:0008152|GO:0008484
		IMPJCBKJ_00307	524	ProSitePatterns	PS00149	Sulfatases signature 2.	158	168	-	IPR024607	Sulfatase, conserved site	
PsSUL2 S1_4	NHH87776.1	IMPJCBKJ_00303	780	ProSitePatterns	PS00523	Sulfatases signature 1.	109	121	-	IPR024607	Sulfatase, conserved site	
		IMPJCBKJ_00303	780	Pfam	PF00884	Sulfatase	65	479	2,00E-84	IPR000917	Sulfatase, N-terminal	GO:0008152|GO:0008484
PsSUL3 S1_13	NHH87965.1	IMPJCBKJ_00492	498	Pfam	PF00884	Sulfatase	23	352	3,00E-62	IPR000917	Sulfatase, N-terminal	GO:0008152|GO:0008484
PsSUL1 S1_13	NHH90161.1	IMPJCBKJ_02757	514	Pfam	PF00884	Sulfatase	25	371	8.9E-068	IPR000917	Sulfatase, N-terminal	GO:0008152|GO:0008484
PsSUL4 S1_13	NHH89963.1	IMPJCBKJ_02557	508	Pfam	PF00884	Sulfatase	24	356	7,00E-63	IPR000917	Sulfatase, N-terminal	GO:0008152|GO:0008484
	NHH91072.1	IMPJCBKJ_03684	541	Pfam	PF00884	Sulfatase	237	521	7.2E-045	IPR000917	Sulfatase, N-terminal	GO:0008152|GO:0008484
	NHH91071.1	IMPJCBKJ_03683	647	Pfam	PF00884	Sulfatase	279	552	1.1E-035	IPR000917	Sulfatase, N-terminal	GO:0008152|GO:0008484

Supplementary Table 5. Sulfatases for heterologous expression in *E. coli*, features, molecular weight.

Enzyme name/ accession	Organism	Features[a]	bp	Length (aa)[b]	Molecular weight (kDa)
PsSUL1	*Pseudoalteromonas sp.*	His_6 (N-term)	1482	494	57
PsSUL2	*Pseudoalteromonas sp.*	His_6 (N-term)	2271	757	90
PsFucS1	*Pseudoalteromonas sp.*	His_6 (N-term)	1521	507	57
PsSUL3	*Pseudoalteromonas sp.*	His_6 (N-term)	1440	480	55
PsSUL4	*Pseudoalteromonas sp.*	His_6 (N-term)	1467	489	56
Ps3148	*Pseudoalteromonas sp.*	His_6 (N-term)	3117	1039	116
ARS/P51691	*Pseudomonas aeruginosa PAO1*	His_6 (N-term)	1611	537	61
Ary423/AKL72071	*Flammeovirga pacifica*	His_6 (N-term)	1533	511	56

[a] Wild type signal peptide had been removed for codon-optimized synthesized construct.

[b] Including the his-tags

Supplementary Table 6. Crystal structure data collection and refinement statistics.

	PsFucS1
**Data collection**	
Space group	$P2_12_12_1$
Cell dimensions	
a, b, c (Å)	86.20; 182.34; 209.85
α, β, γ (º)	α = β = γ = 90º
Resolution (Å)	48.34-2.50 (2.54-2.50)
R_{pim}	0.105 (0.551)
No. unique reflections	115,081 (78,787)
$I/\sigma I$	8.5 (2.3)
CC (½)	0.991 (0.691)
Completeness (%)	100.0 (100.0)
Redundancy	13.6 (14.0)
**Refinement**	
Resolution (Å)	46.66-2.50
R_{work} / R_{free}	17.16/21.04
No. atoms	
Protein	46,889
Ligand/ion	18
Water	1,330
B-factors (Å²)	
Protein	32.32
Ligand/ion	31.35
Water	29.35
R.m.s deviations	
Bond lengths (Å)	0.003
Bond angles (°)	0.595
**PDB ID**	**7AJ0**

*Values in parentheses are for highest-resolution shell.
